# Activation of ALOX12 by a multi-organelle-orienting photosensitizer drives ACSL4-independent cell ferroptosis

**DOI:** 10.1038/s41419-022-05462-9

**Published:** 2022-12-14

**Authors:** Xiuxia Wang, Yuanhong Chen, Xiang Yang, Lianghui Cheng, Zhenyan He, Yanru Xin, Shan Huang, Fanling Meng, Peijing Zhang, Liang Luo

**Affiliations:** 1 Henan Institute of Medical and Pharmaceutical Sciences, Zhengzhou University, Zhengzhou, 450052 China; 2 National Engineering Research Center for Nanomedicine, College of Life Science and Technology, Huazhong University of Science and Technology, Wuhan, 430074 China; 3 Key Laboratory of Molecular Biophysics of Ministry of Education, College of Life Science and Technology, Huazhong University of Science and Technology, Wuhan, 430074 China; 4 Hubei Key Laboratory of Bioinorganic Chemistry and Materia Medica, School of Chemistry and Chemical Engineering, Huazhong University of Science and Technology, Wuhan, 430074 China

**Keywords:** Cancer metabolism, Cancer metabolism

## Abstract

Ferroptosis is a recently-defined tumor suppression mechanism, but the sensitivity of many tumorigenic cells to ferroptosis is limited by their deficient expression of acyl-CoA synthetase long-chain family member 4 (ACSL4). Here, we report the discovery of a photosensitizer, namely TPCI, which can evoke ACSL4-independent ferroptosis of cancer cells in photodynamic therapy. Through co-localization with 12-lipoxygenase (ALOX12) in multiple subcellular organelles, TPCI activates ALOX12 to generate lipid reactive oxygen species in large quantity and trigger cell ferroptosis. Intriguingly, confining TPCI exclusively in lysosomes switches the cell death from ferroptosis to apoptosis. More strikingly, the ferroptosis mediated by TPCI-induced ALOX12 activation does not require the participation of ACSL4. Therefore, our study identifies TPCI as the first ALOX12 activator to induce ferroptosis independent of ACSL4, which renders a viable therapeutic approach on the basis of distinct ferroptosis of cancer cells, regardless their ACSL4 expressions.

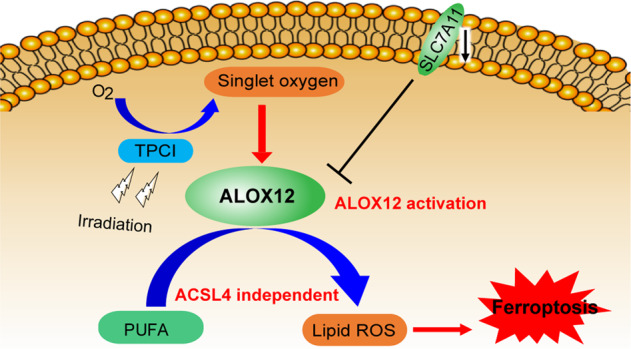

## Introduction

Ferroptosis, a recently identified programmed cell death that occurs via the lethal accumulation of lipid reactive oxygen species (ROS), in an iron-dependent manner, is morphologically, genetically, and biochemically distinct from other forms of regulated cell death [1]. The important role of ferroptosis in suppressing cancer growth and progression has been increasingly recognized [2], given its tremendous potential in eradicating cancer cells with acquired or intrinsic resistance to apoptosis [3], and a number of efforts have been made to design and develop ferroptosis-promoting anticancer drugs [2, 4]. In canonical ferroptosis mediated by glutathione peroxidase 4 (GPX4), the ferroptotic sensitivity of a cell is dictated by Acyl-CoA synthetase long-chain family member 4 (ACSL4) [5], which is an essential contributor to the peroxidation of polyunsaturated fatty acids (PUFAs). However, a large variety of cancer cells have insufficient or downregulated expression of ACSL4, so that they are resistant to GPX4-mediated ferroptosis [5, 6]. Although modulation of ACSL4 may help to sensitize ferroptosis, the situation is usually complicated because the canonical ACSL4 and GPX4-related pathways are vulnerable to the heterogeneity of cancers. An ACSL4-independent ferroptosis is urgent needed to directly benefit the suppression of most cancer cells regardless their ACSL4 expression levels. Recently, activation of arachidonate 12-lipoxygenase (ALOX12), one isoform in the mammalian lipoxygenase family, has been identified to be critical for p53-mediated ferroptosis, the process of which is independent of ACSL4 and does not require GPX4 inhibition [7]. Since the intrinsic modulators that contribute to ALOX12 activation remain largely unknown, a designed ALOX12 activator becomes indispensable and hence holds great promise to promote ACSL4-independent ferroptosis of cancer cells.

To approach an efficient ALOX12 activator, photosensitizers in photodynamic therapy (PDT) [8, 9], which generates cytotoxic ROS upon light irradiation, are particularly promising, in that the generated ROS have the potential to transform PUFA into lipid ROS without the participation of ACSL4. Although PDT has already emerged as a pioneering and effective clinical modality for cancer treatment [10], few PDT-induced ferroptosis has been reported until now. Here we report PDT-induced ferroptosis, using a photosensitizer, namely TPCI [11], with ultraefficient generation of singlet oxygen upon visible light irradiation. Through co-localizing with 12-lipoxygenase (ALOX12) in multiple subcellular organelles, especially endoplasmic reticulum (ER) and Golgi apparatus, TPCI activates ALOX12 significantly upon light irradiation, leading to an effective accumulation of lethal lipid ROS. Inhibition of ALOX12 abrogates TPCI-mediated tumor growth suppression in both in vitro and in vivo models. Interestingly, confining the cellular distribution of TPCI to lysosomes, by encapsulating it in liposomes, switches the cell death from ferroptosis to apoptosis. More strikingly, through expression modulation and activity inhibition, we have unveiled that TPCI-induced ferroptosis does not require the participation of ACSL4, and can be occurred on a variety of cancer cells with different ACSL4 expression levels. Therefore, TPCI represents the first ALOX12 activator to trigger ACSL4-independent ferroptosis. Given that ALOX12 usually functions in many types of cancer cells [12], the discovery of TPCI as a photo-induced ALOX12 activator renders a viable therapeutic approach on the basis of distinct ferroptosis of cancer cells, without limiting to their intrinsic ACSL4 expression levels.

## Results

### Identification of a photosensitizer-induced cancer cell ferroptosis

**Fig. 1:**
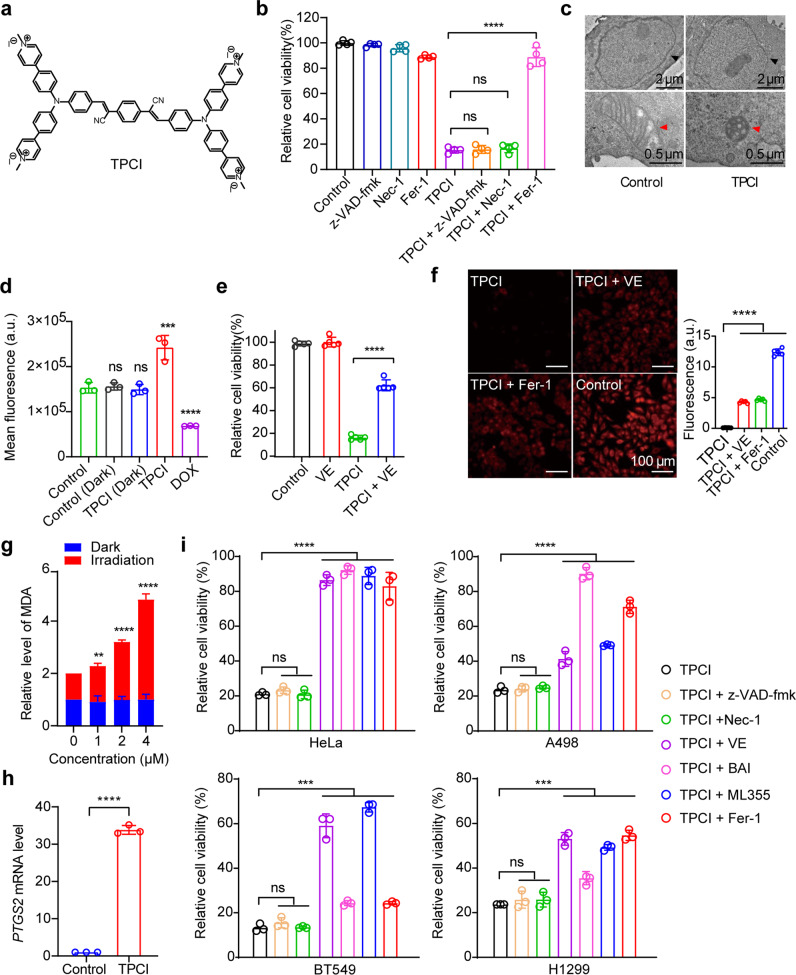
TPCI evokes ferroptosis in human cancer cell lines upon light irradiation. **a** The chemical structure of TPCI. **b** Relative viability of HeLa cells treated by TPCI upon light irradiation, with or without z-VAD-fmk (50 μM), necrostatin-1 (Nec-1, 50 μM), or ferrostatin-1 (Fer-1, 100 μM). **c** Transmission electron microscopy (TEM) images of HeLa cells treated by TPCI upon light irradiation. The black and red arrows referred to cell nucleus and mitochondrion, respectively. **d** Fluorescence of Mito-Tracker Deep Red in HeLa cells treated with DOX (20 μM) or TPCI with light. Data difference were compared to the “Control” group. **e** Relative viability of HeLa cells treated by TPCI upon light irradiation, with or without α-tocopherol (VE, 200 μM). **f** Fluorescence images of HeLa cells treated by TPCI upon light irradiation. The HeLa cells were stained with BODIPY^581/591^ C11 (5 μM), and treated with or without VE (200 μM) or Fer-1 (100 μM). **g** Relative MDA level in HeLa cells treated with various concentrations of TPCI upon light irradiation. Data difference shown were compared to cells treated with TPCI and irradiation. **h** Relative mRNA level of *PTGS2* gene in HeLa cells treated by TPCI upon light irradiation. **i** Relative viability of various cancer cells treated by TPCI upon light irradiation, with or without z-VAD-fmk (50 μM), Nec-1 (50 μM), VE (200 μM), baicalein (BAI, 50 μM), ML355 (30 μM) or Fer-1 (100 μM). TPCI concentration was 1 μM for HeLa cells and A498 cells, while the concentration of TPCI was 3 μM for BT549 cells and H1299 cells; Irradiation conditions: 460 nm, 1 mW cm^−2^, 20 min. The data were shown as mean ± SD from a representative experiment (*n* = 3–5) of 2–3 independent biological replicates. Statistical significance was analyzed by using two-tailed unpaired Student’s *t* test (ns no significance, *p < 0.05, **p < 0.01, ***p < 0.001, and ****p < 0.0001).

When TPCI (Fig. 1a) was delivered through encapsulated nanoparticles, TPCI triggered apoptosis of cancer cells in PDT [13]. Strikingly, the HeLa cells pretreated with the free form of TPCI displayed dramatic cell morphological changes upon visible light irradiation, including cell blebbing but no chromatin condensation and margination, which suggested a non-apoptosis cell death (Supplementary Fig. 1a). In addition, no caspase-3 cleavage, one hallmark of apoptosis, was observed (Supplementary Fig. 1b). The increased uptake of propidium iodide (Supplementary Fig. 1c) indicated the rupture of plasma membranes that were consistent with caspase-independent non-apoptotic cell death.

To identify the form of cell death triggered by TPCI more definitively, various inhibitors targeting different cell death-associated pathways were applied to regulate the viability of HeLa cells. Interestingly, none of z-VAD-fmk (a pan caspase inhibitor) [14], N-phenylmaleimide (an apoptosis-inducing factor inhibitor) [15], or necrostatin-1 (Nec-1, an inhibitor of necroptosis) [16] could rescue HeLa cells from death. In contrast, ferrostatin-1 (Fer-1), a typical inhibitor of ferroptosis [17], recovered the cell viability to ~90% (Fig. 1b and Supplementary Fig. 1d). Careful inspection of the organelle morphology of the dying cells also suggested a form of ferroptosis, involving shrunken mitochondrion with dense membrane as well as intact nucleus with complete structure (Fig. 1c and Supplementary Fig. 1e). Furthermore, we used Mito-Tracker Red to monitor the change of mitochondrial membrane potential (MMP) in cells treated by TPCI, using doxorubicin hydrochloride (DOX) as a control for apoptosis. As expected, DOX decreased MMP, whereas light irradiated TPCI caused MMP hyperpolarization that was commonly seen in ferroptosis (Fig. 1d and Supplementary Fig. 1f) [18]. The equal expressions of LC3-I and LC3-II between HeLa cells treated by TPCI and without any treatment further excluded the cell death from autophagy (Supplementary Fig. 1g) [16]. Collectively, the foregoing data suggested that the cell death triggered by TPCI was independent of apoptosis, necroptosis, or autophagy, but more likely ferroptosis.

In addition, iron chelators could alleviate the cytotoxicity of TPCI upon irradiation (Supplementary Fig. 1h), which coincided with the iron-dependent feature of ferroptosis. α-tocopherol (VE), a lipophilic scavenger of cytoplasmic lipid ROS, reduced the toxicity significantly (Fig. 1e), suggesting the characteristic of lipid ROS-induced ferroptosis [19]. The lipid peroxidation sensor BODIPY^581/591^ C11 was applied to evaluate lipid peroxidation in cells by exploiting its loss of red fluorescence upon interaction with peroxyl radicals [20–23]. HeLa cells pretreated with TPCI exhibited significantly lower red fluorescence signals under light than in dark (Supplementary Figs. 1i, j and 2a). Since BODIPY^581/591^ C11 itself was unaffected under visible light irradiation, the attenuation of red fluorescence signals suggested that abundant lipid ROS were generated in cells. Furthermore, we observed that the red fluorescence of BODIPY^581/591^ C11 was retained in the presence of lipophilic scavengers of ROS (VE and Fer-1) (Fig. 1f and Supplementary Fig. 2b). This result was also consistent with the fact that cells could be rescued by hydrophobic antioxidants. The generation of malonaldehyde (MDA), a secondary product of cellular lipid ROS, was enhanced as the dose of TPCI increased (Fig. 1g), further confirming the generation of lipid ROS. Notably, TPCI also significantly upregulated the expression of *PTGS2*, another marker of ferroptosis [24], in HeLa cells (Fig. 1h). Collectively, these data confirmed that TPCI induced ferroptosis of HeLa cells upon light irradiation.

We next applied TPCI on a series of additional human cancer cell lines, including BT549 cells, H1299 cells, and A498 cells in the presence of various inhibitors (inhibitors showed no obvious cytotoxicity, Supplementary Fig. 2c). As shown in Fig. 1i, all cancer cells treated by TPCI exhibited no response to z-VAD-fmk or Nec-1 under irradiation, and no cleaved caspase-3 was observed in these cancer cells (Supplementary Fig. 2d). Instead, VE, Fer-1, and lipoxygenases inhibitors (baicalein and ML355) were highly protective on all the human carcinoma cell lines. It is noted that the expression levels of ACSL4 varied significantly in these cells (Supplementary Fig. 2e), and the TPCI-induced ferroptosis was applicable to all of these human cancer cell lines.

### TPCI-evoked ferroptosis is regulated by ALOX12

**Fig. 2:**
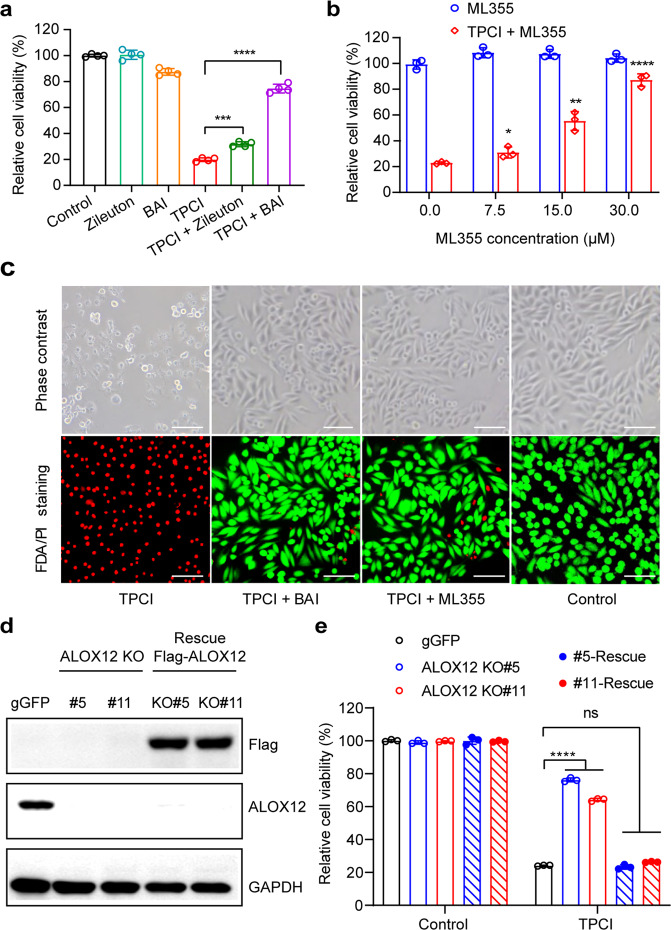
TPCI-induced cell ferroptosis is mediated by ALOX12. **a** Relative viability of HeLa cells treated by TPCI and light irradiation, with or without baicalein (BAI, 50 μM) or zileuton (100 μM). **b** Relative viability of HeLa cells treated by TPCI and light irradiation in the presence of different concentrations of ML355. Data difference shown were compared to cells treated with TPCI and irradiation. **c** Phase contrast microscope images and living/death staining of HeLa cells treated by TPCI and light irradiation, with or without BAI (50 μM) or ML355 (30 μM). Scale bar: 100 μm. **d** Knockout (KO) and rescue of ALOX12 expression in HeLa cells determined by western blotting of ALOX12 or Flag tag. **e** Relative viability of the control CRISPR (gGFP), ALOX12-KO or ALOX12-KO rescue HeLa cells treated by TPCI and light irradiation. TPCI concentration: 1 μM; Irradiation conditions: 460 nm, 1 mW cm^−2^, 20 min. The data were shown as mean ± SD from a representative experiment (*n* = 3–6) of 2–3 independent biological replicates. Statistical significance was analyzed by using two-tailed unpaired Student’s *t* test (ns no significance, *p < 0.05, **p < 0.01, ***p < 0.001, and ****p < 0.0001).

Lipid ROS in ferroptotic cells can be generated by Fenton reaction and/or ALOXs. Meanwhile, TPCI can generate a large amount of ROS, which is required for activating the inactive ferrous form to an active ferric form of ALOX12 [25]. Therefore, we speculate that ALOXs may participate in the TPCI-induced ferroptosis. To examine whether ALOXs [26] were involved in TPCI-mediated ferroptosis, we compared the sensitivity of TPCI-evoked ferroptosis to a series of ALOX inhibitors. Zileuton, an inhibitor of ALOX5 [27], was slightly effective in preventing ferroptotic death triggered by TPCI. However, baicalein, an inhibitor of both ALOX15 and ALOX12 [28], was highly efficient in rescuing cells (Fig. 2a). To ascertain the effect of ALOX15, we knocked down the expression of ALOX15 in HeLa cells (Supplementary Fig. 2f). There was no difference in cell viability between the scramble cells and the shALOX15 cells post the TPCI treatment (Supplementary Fig. 2g), suggesting that ALOX15 was not indispensable to TPCI-triggered ferroptosis. We next employed ML355, an exclusive inhibitor of ALOX12 [29], to study the ALOX12 function in TPCI-induced ferroptosis. Strikingly, ML355 rescued cell from death in a dose-dependent manner. When the concentration of ML355 reached 30 μM, the cell viability could be recovered from 20% to 90% (Fig. 2b, c). The generation of cellular lipid ROS was significantly reduced as well (Supplementary Fig. 2h), suggesting that the activity of ALOX12 was pivotal to TPCI-evoked cell ferroptosis. To further elucidate ALOX12 participation in the ferroptotic death caused by TPCI, we used clustered regularly interspaced short palindromic repeats (CRISPR)-associated protein 9 (Cas9) technology to generate ALOX12-knockout subclones of HeLa cells (Fig. 2d). As expected, the ALOX12-knockout cells were resistant to ferroptosis induced by TPCI (Fig. 2e). Of note, we recovered the expression of ALOX12 in ALOX12-knockout HeLa cells, where the sensitivity to TPCI were restored (Fig. 2e). In addition, ALOX12-knockdown HeLa cells (Supplementary Fig. 2i, j) were insensitive to TPCI-induced ferroptosis and exhibited less lipid peroxidation than the scramble HeLa cells (Supplementary Fig. 2k–m), while the sensitivity to TPCI and the accumulation of lipid ROS could be restored when ALOX12 rescue in HeLa cells (Supplementary Fig. 2n, o). Taken together, these data indicated that ALOX12 regulated TPCI-evoked cell ferroptosis through influencing the generation of lipid ROS.

### Inactivation of ALOX12 abrogates TPCI-mediated tumor growth suppression

**Fig. 3:**
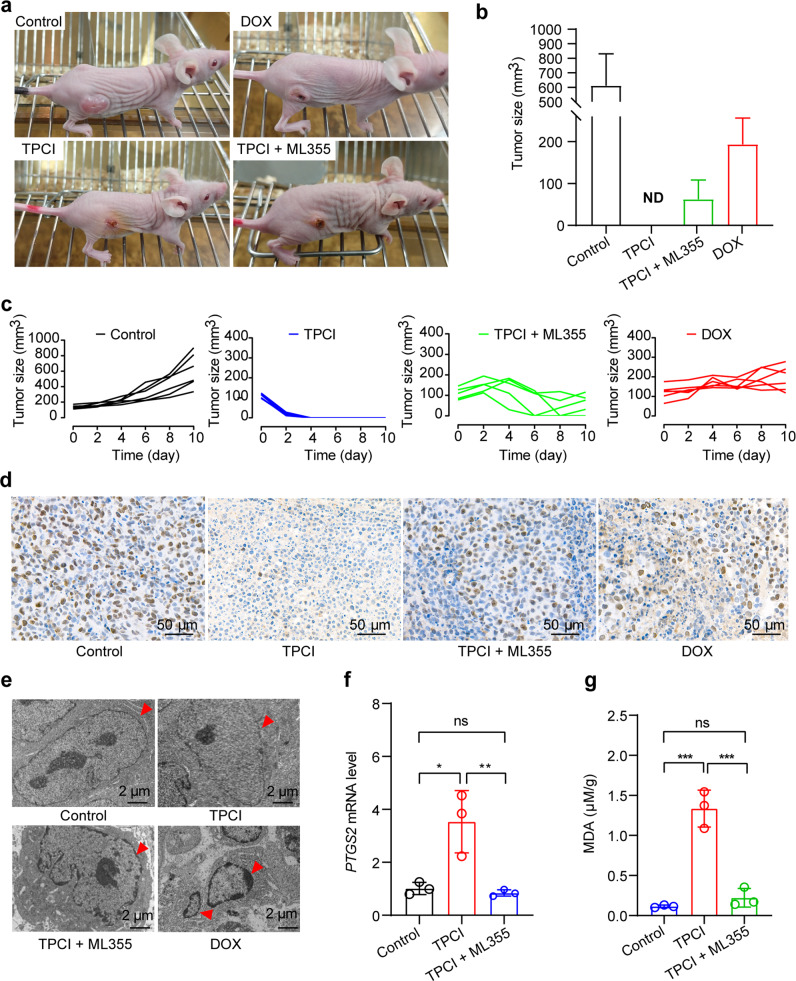
ALOX12 is critical to TPCI-induced ferroptosis in vivo. **a** Photographs of representative tumor-bearing mice on Day 10 post the treatment by TPCI (“TPCI”, *n* = 6), TPCI and ML355 (“TPCI + ML355”, *n* = 5), doxorubicin hydrochloride (“DOX”, *n* = 6), and saline (“Control”, *n* = 6). **b** The average tumor sizes of mice in different groups on Day 10 post treatment. **c** Individual tumor growth kinetics. **d** Representative histological examinations of the dissected tumors on Day 2 post treatment using Ki67 staining. **e** Representative TEM images of dissected tumors on Day 2 post treatment from various groups. The red arrows referred to cell nuclei. **f** mRNA expression levels of *PTGS2* gene in tumors harvested on Day 2 post treatment (*n* = 3). **g** The MDA content of tumors harvested on Day 2 post treatment (*n* = 3). The data were shown as mean ± SD; Statistical significance was analyzed by using two-tailed unpaired Student’s *t* test (ns no significance, *p < 0.05, **p < 0.01, ***p < 0.001, and ****p < 0.0001).

To confirm the contribution of ALOX12 to the tumor suppression activity of TPCI, we tested whether inhibiting ALOX12 activity could affect TPCI-mediated tumor cell growth on HeLa-cells-xenografted nude mice model. TPCI significantly suppressed tumor cell growth in this assay when exposed to light irradiation (Supplementary Fig. 3a–c and Fig. 3a), and eliminated the tumor on Day 4 post treatment. However, inhibition of ALOX12 activity by ML355 diminished the anti-tumor efficiency of TPCI (Fig. 3b, c). In addition, the TPCI group showed largest tumor lesion in H&E staining (Supplementary Fig. 3d) among all testing groups, as well as massive cell death without apoptosis maker caspase-3 activation (Supplementary Fig. 3e) or proliferation marker anti-Ki67 (Fig. 3d). Moreover, tumor cells in the TPCI group showed shrunken mitochondria with dense membrane (Supplementary Fig. 3f) and intact nucleus with complete structure (Fig. 3e), consistent with the cell ferroptosis. Notably, upregulation of the ferroptosis marker *PTGS2* was also abrogated in ML355-containing group (Fig. 3f), implying that inhibiting ALOX12 revoked TPCI-induced ferroptosis. Because lipid peroxidation is a key feature in ferroptosis, we lastly checked the MDA content in tumors. TPCI significantly enhanced the MDA level in tumors, while ML355 dramatically diminished MDA (Fig. 3g). In addition, we silenced the mRNA of ALOX12 using shRNAs that caused the knockdown of ALOX12 in human lung carcinoma cells (H1299) and human renal clear cell carcinoma cells (786-O) (Supplementary Fig. 3g). As expected, both of the ALOX12-knockdown cancer cells were resistant to ferroptosis induced by TPCI upon irradiation (Supplementary Fig. 3h, i). Collectively, the above results confirmed that ALOX12 was indispensable to TPCI-induced cell ferroptosis.

### Activation of ALOX12 by TPCI

**Fig. 4:**
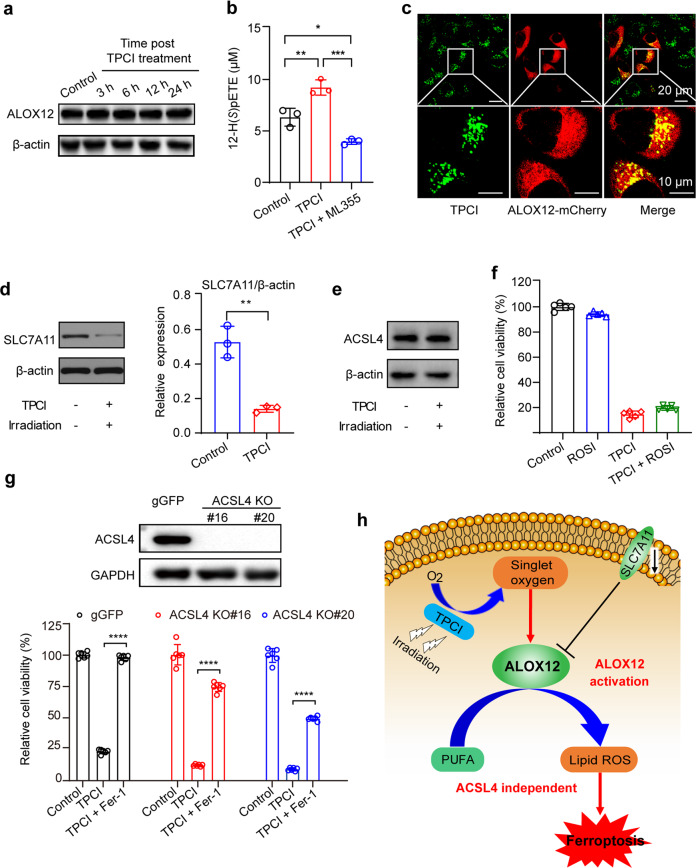
TPCI-induced ALOX12 activation triggers ACSL4-independent ferroptosis. **a** ALOX12 expression of HeLa cells at different time post the TCPI treatment. **b** The concentration of 12-H(*S*)pETE in the mixture of recombinant ALOX12 and arachidonic acid at different conditions. TPCI was 5 μM and ML355 was 10 μM. **c** The confocal laser scanning microscopy (CLSM) images ALOX12-mCherry-expressed HeLa cells stained by TPCI (1 μM). **d** SLC7A11 and p53 expression of HeLa cells with and without the TPCI treatment. **e** ACSL4 expression of HeLa cells at 6 h post the TPCI treatment. **f** Relative viability of HeLa cells receiving the TPCI treatment in the presence of ROSI (20 μM). **g** Relative viability of the control CRISPR (gGFP) or ACSL4-knockout (KO) HeLa cells treated by TPCI and light irradiation with or without Fer-1 (100 μM for 6 h); Inset: Knockout of *ACSL4* gene in HeLa cells determined by western blotting of ACSL4. **h** Schematic illustration of TPCI-induced activation of ALOX12 upon light irradiation to evoke ACSL4-induced ferroptosis. All irradiation conditions: 460 nm, 1 mW cm^−2^, 20 min. The data were shown as mean ± SD from 3–6 independent biological replicates. Statistical significance was analyzed by two-tailed unpaired Student’s *t* test (*p < 0.05, **p < 0.01, ***p < 0.001, and ****p < 0.0001).

The crucial role of ALOX12 in TPCI-triggered cell ferroptosis inspired us to investigate how ALOX12 was modulated by TPCI. Notably, the expression level of ALOX12 in HeLa cells remained constant after the TPCI treatment for up to 24 h (Fig. 4a). We then studied the lipoxygenase activity of a recombinant ALOX12 (Supplementary Fig. 3j, k) in the presence of arachidonic acid and TPCI. Upon irradiation, the peroxidation of arachidonic acid to (12S)-hydroperoxyeicosatetraenoate (12-H(*S*)pETE) was significantly enhanced (Fig. 4b), indicating the activation of ALOX12 by TPCI. As a comparison, the production of 12-H(*S*)pETE almost remained unchanged without irradiation (Supplementary Fig. 3l). In addition, TPCI alone, in the absence of ALOX12, could not induce the peroxidation of arachidonic acid, regardless irradiation (Supplementary Fig. 3m). Therefore, ALOX12 was directly activated by the TPCI-generated ROS upon irradiation. We further studied the co-localization of TCPI and ALOX12 by fluorescence imaging of living HeLa cells, in which ALOX12 was fusion expressed with a red fluorescent protein (mCherry) tag. After being incubated with TPCI (1 μM) in dark for 24 h, the fluorescence images of HeLa cells suggested that the ingested TPCI mostly resided in the ALOX12 adjacency (Fig. 4c). Since the ROS-causing damage was confined within the vicinity of the photosensitizer due to limited lifetime and migration distance of radicals [30, 31], the close contact between TPCI and ALOX12 revealed the potential of TPCI in activating ALOX12 by its generated ROS in cells.

Surprisingly, we have found that TPCI downregulated the expression of SLC7A11 (Fig. 4d and Supplementary Fig. 3n, o) upon irradiation. SLC7A11 has been identified as a bona fide binding partner of ALOX12 [7], and the binding between SLC7A11 and ALOX12 inhibits the enzymatic activity of ALOX12. Therefore, the generated ROS by TPCI suppressed the expression of SLC7A11 and restored the activity of ALOX12. However, the expression of p53 exhibited negligible change in the process (Supplementary Fig. 3p). Given that p53 has been reported to regulate the SLC7A11 expression [32], our results indicated that TPCI activated ALOX12 through SLC7A11 in a different manner.

### TPCI-induced ferroptosis is independent of ACSL4

Recent studies have established that ACSL4 is an essential biomarker and contributor of ferroptosis, and dictates the sensitivity of a cell to ferroptosis [5, 6]. However, we observed that the expression level of ACSL4 in HeLa cells was not affected by the TPCI treatment (Fig. 4e). Moreover, rosiglitazone (ROSI), a typical inhibitor of ACSL4, was slightly effective in rescuing TPCI-induced cell death (Fig. 4f). In addition, TPCI-induced ferroptosis was not suppressed in ACSL4-knockout HeLa cells (Fig. 4g), while the ACSL4-knockout HeLa cells were resistant to ferroptosis induced by a classic ferroptosis inducer named RSL3 (Supplementary Fig. 3q). However, Fer-1 could also recover the viability of ACSL4-knockout HeLa cells treated with TPCI and light irradiation significantly (Fig. 4g). Meanwhile, we knocked down the expression of ACSL4 in HeLa cells by RNA interference (Supplementary Fig. 3r). None of these ACSL4 knockdown could prevent HeLa cells from TPCI-induced ferroptosis (Supplementary Fig. 3s). Taken together, these data indicate that TPCI-induced ferroptosis is independent of ACSL4.

Collectively, the foregoing data demonstrated that TPCI generated ROS upon light irradiation, which either directly activated ALOX12 or resurged ALOX12 via SLC7A11 downregulation. The TPCI-induced ALOX12 activation resulted in direct peroxidation of PUFAs into lethal lipid ROS, which then accumulated and triggered cancer cells ferroptosis without the participation of ACSL4 (Fig. 4h).

### Distribution of photosensitizers affects the cell death type

As shown in Fig. 4b, TPCI regulates the activity of ALOX12 directly upon irradiation. We used in silico method to investigate the binding of ALOX 12 with TPCI and other photosensitizers. Computational modeling showed that ALOX12 could bind with a variety of photosensitizers. The binding affinity of ALOX12 with TPCI and its analogue TPBT were higher than with other photosensitizers, such as protoporphyrin (PpIX), rose bengal (RB), and methylene blue (MB). Moreover, TPCI and TPBT shared the same docking sites in ALOX12, given their similar chemical structures. Interestingly, TPCB, another analogue of TPCI but with terminal ammonium groups, showed lower binding constant, indicating that the pyridinium groups in TPCI and TPBT assisted their binding with ALOX12 (Supplementary Fig. 4a, b). We next examined whether other photosensitizers could induce ferroptosis of HeLa cells upon irradiation. As shown in Supplementary Fig. 4c, TPCI and TPBT showed similar pharmacological characteristics to induce cell ferroptosis post irradiation, whereas apoptosis inhibitor z-VAD-fmk and hydrophilic antioxidant sodium ascorbate (VC, the inhibitor of ROS-induced apoptosis [33]) inhibited cell death induced by other three commercial photosensitizers in PDT. Moreover, MB and RB treatment triggered the production of cleaved caspase-3 (Supplementary Fig. 4d). On the contrary, we observed that the ferroptosis markers *PTGS2* and *LPCAT3* only slightly increased in MB treatment, in contrast to the substantial enhancement in TPCI treatment (Supplementary Fig. 4e, f). Remarkably, modulating the expression of ALOX12 in HeLa cells had no obvious effect on the anti-tumor efficiency of MB treatment (Supplementary Fig. 4g, h), suggesting that ALOX12 did not contribute to the MB treatment-induced apoptosis. In addition, the cell death process caused by MB and RB treatment was independent of GPX4 (Supplementary Fig. 4i–l), further confirming that these photosensitizers induced cell apoptosis rather than ferroptosis in PDT.

**Fig. 5:**
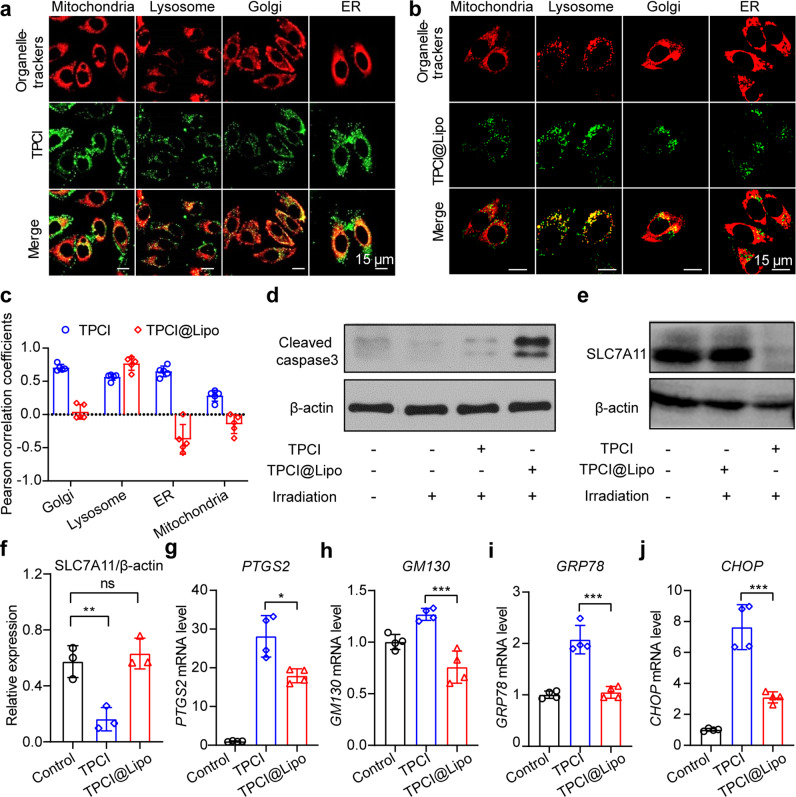
The distribution of TPCI affects the manner of cell death. **a**, **b** Subcellular co-localization of TPCI (1 μM) and TPCI@Lipo (containing 1 μM of TPCI) with different organelle trackers, including Mito-Tracker Deep Red FM, Lyso-Tracker Red DND, Golgi-Tracker Red and ER-Tracker Red. **c** Pearson correlation coefficients of TPCI and different organelle trackers. All data were represented as mean ± SD, *n* = 5 independent fields. **d** Cleaved caspase-3 and β-actin expression in HeLa cells with TPCI or TPCI@Lipo treatment. **e**, **f** SLC7A11 expression of HeLa cells with TPCI or TPCI@Lipo treatment. Data were showed as mean ± SD from three independent experiments. Relative mRNA level of *PTGS2* (**g**), *GM130* (**h**), *GRP78* (**i**), and *CHOP* (**j**) in HeLa cells receiving TPCI or TPCI@Lipo treatment compared to untreated cells. TPCI or TPCI@Lipo treatment: TPCI concentration: 1 μM; All irradiation conditions: 460 nm, 1 mW cm^−2^, 20 min. The data were showed as mean ± SD from a representative experiment (*n* = 3–4) out of three independent biological replicates. Statistical significance was analyzed by two-tailed unpaired Student’s *t* test (ns no significance, *p < 0.05, **p < 0.01, ***p < 0.001, and ****p < 0.0001).

The different cell death types in PDT triggered by TPCI and by other photosensitizers urged us to examine the intracellular distribution of TPCI in HeLa cells. PDT has been discovered to promote apoptosis, autophagy, or necrosis response of malignant cells. However, PDT-induced ferroptosis has been scarcely reported. This can be explained by the short lifetime and nanometer migration distance of the generated ROS in PDT, which only results in the damages within the vicinity of the photosensitizers. In addition, the cellular distributions of photosensitizers affect the efficiency and phenotype of cell death in PDT [34, 35]. After co-incubating HeLa cells with TPCI (1 μM) for 24 h, four commercial fluorescent probes, Golgi-Tracker Red, ER-Tracker Red, Mito-Tracker Deep Red, and Lyso-Tracker Red, were employed to stain HeLa cells respectively. As illustrated in Fig. 5a and Supplementary Fig. 5a–d, TPCI co-localized with all these dyes in HeLa cells, with Pearson’s correlation coefficients of 0.71 (for Golgi), 0.65 (for ER), 0.56 (for lysosomes), and 0.28 (for mitochondria), indicating that TPCI accumulated in all of these subcellular organelles, especially in Golgi apparatus and ER (Fig. 5c). Since both Golgi oxidative stress and ER oxidative stress have been demonstrated to mediate redox imbalance and ferroptosis [36, 37], we hypothesized that TPCI induced oxidative stresses in Golgi apparatus and ER, which may sensitize HeLa cells to ferroptosis. To this end, we encapsulated TPCI into nanoparticles with diameters of around 100 nm by liposomes (TPCI@Lipo). Once ingested by HeLa cells, TPCI@Lipo co-localized with the Lyso-Tracker Red exclusively, with a Pearson’s correlation coefficient of over 0.75, indicating that TPCI@Lipo mainly resided in lysosomes (Fig. 5b, c and Supplementary Fig. 5e–h). Markedly, restriction of TPCI in lysosomes increased caspase-3 cleavage (Fig. 5d), switching cell death to caspase-dependent apoptosis. In addition, we observed that the SLC7A11 expression in HeLa cells with TPCI@Lipo treatment kept stable, in contrast to the sharp decrease in cells with TPCI treatment (Fig. 5e, f). In addition, *PTGS2* gene expression was significantly lower in HeLa cells treated by TPCI@Lipo than by TPCI (Fig. 5g). Particularly, TPCI treatment induced oxidative stresses to both Golgi and ER, as evidenced by the upregulation of Golgi apparatus oxidative stress related genes *GM130* and ER oxidative stress related genes *CHOP* and *GRP78* (Fig. 5h–j). However, the impact on the expression of these genes were much less significant in cells receiving TPCI@Lipo treatment, suggesting that the phenotype of cell death in PDT was directly correlated to the organelle distribution of TPCI.

### GPX4 is inactivated by TPCI

**Fig. 6:**
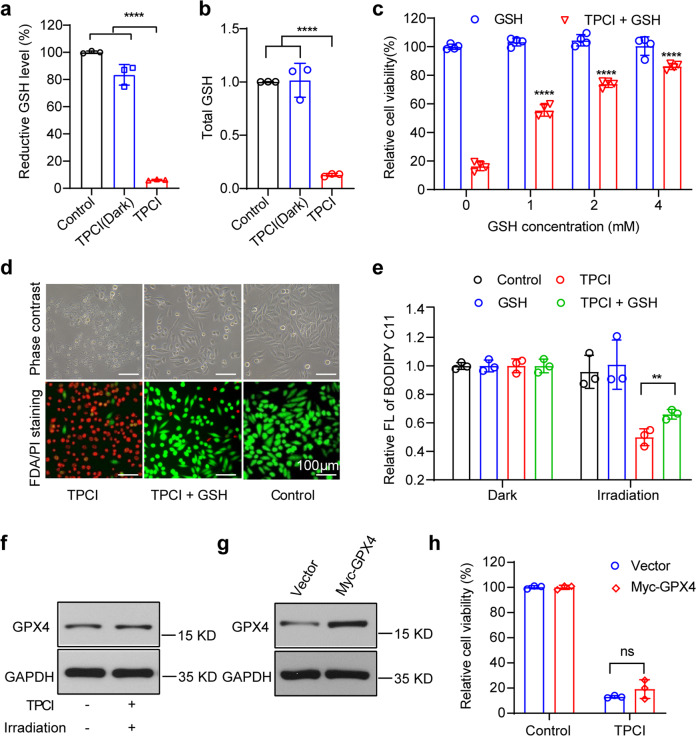
GPX4 is indirectly inactivated by TPCI. The reductive GSH (**a**) and total GSH (**b**) levels of HeLa cells treated by TPCI and light irradiation (data from three independent biological replicates). **c** Relative viability of HeLa cells treated by TPCI and light irradiation, with or without various concentrations of GSH. **d** Phase contrast microscope images and FDA/PI staining of HeLa cells treated by TPCI and light irradiation, with or without GSH (4 mM). **e** Relative fluorescence intensity of BODIPY^581/591^ C11 in HeLa cells treated by TPCI and light irradiation, with or without GSH (4 mM). **f** GPX4 expression of HeLa cells treated by TPCI and light irradiation. **g** Overexpression of GPX4 in HeLa cells (Myc-GPX4). **h** Relative viability of the control and GPX4-overexpressed HeLa cells treated by TPCI and light irradiation (data from three independent biological replicates). TPCI concentration was 1 μM. All irradiation conditions: 460 nm, 1 mW cm^−2^, 20 min. The data were shown as mean ± SD from a representative experiment (*n* = 3–4) from of 2–3 independent biological replicates. Statistical significance was analyzed by two-tailed unpaired Student’s *t* test (ns no significance, *p < 0.05, **p < 0.01, ***p < 0.001, and ****p < 0.0001).

Since SLC7A11 is a cystine/glutamate antiporter that promotes intracellular cystine uptake to synthesize GSH, an essential cofactor of GPX4, the deduction of SLC7A11 in HeLa cells by irradiated TPCI inspired us to examine whether TPCI affected GSH levels, as well as the capability of GPX4 in depleting lipid ROS, in PDT. When irradiated, TPCI triggered 90% deduction of the total GSH in HeLa cells, and more than 94% of reductive GSH was depleted, along with a 3.5-fold increase of oxidative GSSG (Fig. 6a, b and Supplementary Fig. 6a). Therefore, TPCI could inactivate GPX4 through GSH depletion, which allowed for excessive lipid ROS accumulation in cells. In fact, the cell viability was significantly increased with the supplement of exogenous GSH, in a dose-dependent manner (Fig. 6c), which also improved the survival of dying cells (Fig. 6d) through the diminishment of lipid ROS (Fig. 6e and Supplementary Fig. 6b). Interestingly, TPCI did not change the expression of GPX4 in HeLa cells upon irradiation (Fig. 6f), and overexpressing GPX4 (Fig. 6g) could not rescue cells from TPCI-induced ferroptosis (Fig. 6h). In addition, the viability of the GPX4-knockdown HeLa cells was also not affected (Supplementary Fig. 6c, d). The weak impact of GPX4 overexpression on cell ferroptosis suggested that TPCI-triggered GSH depletion blocked the function of GPX4, further confirming that TPCI-induced ferroptosis was executed through both ALOX12 activation and GPX4 inhibition. The existing ferroptosis-inducing drugs (such as erastin and RSL3) that rely on GPX4 inhibition are only effective on a small portion of cancers, due to the limited lipid ROS generation. As a comparison, TPCI not only inhibits GPX4 pathway, but more importantly, is capable of generating a large amount of lipid ROS through ALOX12 activation, so that it holds great potential to be universally applicable on the treatment of a large variety of cancers.

## Discussion

PDT can reinforce ferroptosis induction in anticancer therapy [38], especially when used in combination with inducers of ferroptosis [39]. In addition, almost all recent findings of PDT-induced ferroptosis are through the canonical ACSL4 and GPX4-related pathways [40, 41], which are vulnerable to the heterogeneity of cancers. Although the precise mechanism of TPCI-induced ferroptosis still requires further elucidation, our study emphasizes the significance of ALOX12 activation in this process, which enables ferroptosis independent of ACSL4. Ferroptosis is recently highlighted with clinical significance for tumor treatments, because it can overcome inevitable barriers of the currently prevalent apoptosis-mediated therapy. However, increased studies have evidenced that ferroptosis by those canonical inducers (e.g., erastin, sorafenib, RSL3, fluvastatin) have resistance problems on cells with low expression of ACSL4 [42]. We have showed that TPCI can activate ALOX12 upon irradiation and enhance lipid ROS accumulation, hence promoting ferroptosis of cancer cells that does not require ACSL4. As ALOX12 is highly expressed in cancer cells [43–46] and no ALOX12 activator has been reported so far, our study unveiling TPCI-induced ALOX12 activation should broaden the applicability of ferroptosis in cancer therapy. On the other hand, our results indicate a new paradigm concerning the fate of cells challenged by photosensitization. The concept of modulating oxidative stress in multiple organelles, as well as switching cell death type by adjusting the cellular distribution of photosensitizers, will allow new strategies to overcome acquired resistance to cell death, inspiring the development of more efficient drugs against cancer as well as other proliferative diseases.

## Supplementary information

aj-checklist

Original Data File

Supplementary Information

## Data Availability

The data analyzed and used to support this study can be found within the main text, supporting information. All other data are available from the corresponding authors upon reasonable request.
