# Supplementary material for: Activation of ALOX12 by a multi-organelle-orienting photosensitizer drives ACSL4-independent cell ferroptosis
Source: Cell Death Dis. 2022 Dec 14;13(12):1040. doi: 10.1038/s41419-022-05462-9 (PMC9751149; doi:10.1038/s41419-022-05462-9)
Supplement: Supplementary file 1 — aj-checklist [file 41419_2022_5462_MOESM1_ESM.docx]

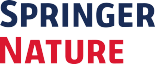


**Corresponding** **Author** **Name:** Liang Luo

**Manuscript** **Number:** CDDIS-22-2567RRR

Reporting Summary

***Springer*** ***Nature*** ***wishes*** ***to*** ***improve*** ***the*** ***reproducibility*** ***of*** ***the*** ***work*** ***that*** ***we*** ***publish.*** ***This*** ***checklist*** ***is*** ***used*** ***to*** ***ensure*** ***good*** ***reporting*** ***standards*** ***and*** ***to*** ***improve*** ***the*** ***reproducibility.*** ***Please*** ***respond*** ***completely*** ***to*** ***all*** ***questions*** ***relevant*** ***to*** ***your*** ***manuscript.*** ***For*** ***more*** ***information,*** ***please*** ***read*** ***the*** ***journal’s*** ***Guide*** ***to*** ***Authors.***

☑ **Check** **here** **to** **confirm** **that** **the** **following** **information** **is** **available** **in** **the** **Material** **&** **Methods** **section:**


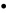
 **The** **exact** **sample** **size** **(*n)*** for each experimental group/condition, given as a number, not a range


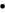
 **A** **description** **of** **the** **sample** **collection** allowing the reader to understand whether the samples represent

technical or biological replicates (including how many animals, litters, culture, etc.)


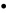
 **A** **statement** **of** **how** **many** **times** **the** **experiment** **shown** **was** **replicated** **in** **the** **laboratory**
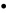
 **Definitions** **of** **statistical** **methods** **and** **measures**: For small sample sizes (n<5) descriptive statistics are not

appropriate, instead plot individual data points

o Very common tests, such as *t*-test, simple χ2 tests, Wilcoxon and Mann-Whitney tests, can be unambiguously identified by name only, but more complex techniques should be described in the methods section

o Are tests one-sided or two-sided?

o Are there adjustments for multiple comparisons?

o **Statistical** **test** **results**, e.g., ***P*** **values**

o Definition of **‘center** **values’** as **median** **or** **mean**;

o Definition of **error** **bars** **as** **s.d.** **or** **s.e.m.** **or** **c.i.**

***Please*** ***ensure*** ***that*** ***the*** ***answers*** ***to*** ***the*** ***following*** ***questions*** ***are*** ***reported*** ***in*** ***the*** ***manuscript*** ***itself.*** ***We*** ***encourage*** ***you*** ***to*** ***include*** ***a*** ***specific*** ***subsection*** ***in*** ***the*** ***methods*** ***section*** ***for*** ***statistics,*** ***reagents*** ***and*** ***animal*** ***models.*** ***Below,*** ***provide*** ***the*** ***page*** ***number*** ***or*** ***section*** ***and*** ***paragraph*** ***number.***

Statistics and general methods

1. How was the sample size chosen to ensure adequate power to detect a pre-specified effect size? (Give section/paragraph or page #)

For animal studies, include a statement about sample size estimate even if no statistical methods were used.

2. Describe inclusion/exclusion criteria if samples or animals were excluded from the analysis. Were

the criteria pre-established? (Give

section/paragraph or page #)

3. If a method of randomization was used to determine how samples/animals were allocated to experimental groups and processed, describe it. (Give section/paragraph or page #)

For animal studies, include a statement about

randomization even if no randomization was used.

**Reported** **in** **section/paragraph** **or** **page** **#**

| Section “Figure Legends” |
| --- |
| Experimental Methods section “In Vivo Antitumor Efficacy” (page S16) |
| n/a |
| Experimental Methods section “In Vivo Antitumor Efficacy” (page S16) |
| Experimental Methods section “In Vivo Antitumor Efficacy” (page S16) |

*1*

4. If the investigator was blinded to the group allocation during the experiment and/or when assessing the outcome, state the extent of blinding. (Give section/paragraph or page #)

For animal studies, include a statement about

blinding even if no blinding was done.

5. For every figure, are statistical tests justified as appropriate?

Do the data meet the assumptions of the tests (e.g., normal distribution)?

Is there an estimate of variation within each group of data?

Is the variance similar between the groups that are

being statistically compared? (Give

section/paragraph or page #)

Reagents

6. Report the source of antibodies (vendor and catalog number)

7. Identify the source of cell lines and report if they were recently authenticated (e.g., by STR profiling) and tested for mycoplasma contamination

Animal Models

8. Report species, strain, sex and age of animals

9. For experiments involving live vertebrates, include a statement of compliance with ethical regulations and identify the committee(s) approving the experiments.

| n/a |
| --- |
| Experimental Methods section “In Vivo Antitumor Efficacy” (page S16) |
| Yes, Reported in Experimental Methods section “Statistical Analysis” (page S17) |
| Yes, Reported in Experimental Methods section “Statistical Analysis” (page S17) |
| Yes, Reported in Experimental Methods section “Statistical Analysis” (page S17) |
| Yes, Reported in Experimental Methods section “Statistical Analysis” (page S17) |

Reported in section/paragraph or page #

| Yes, Reported in Experimental Methods section “Western Blot (WB) Analysis” (page S4 and page S5) |
| --- |
| Reported in Experimental Methods section “Cell Culture” (page S3) |

Reported in section/paragraph or page #

| Reported in Experimental Methods section “In Vivo Antitumor Efficacy” (page S16) |
| --- |
| Reported in Experimental Methods section “In Vivo Antitumor Efficacy” (page S16) |

10. We recommend consulting the ARRIVE guidelines [(*PLoS* *Biol.* 8(6), e1000412,2010](http://www.ncbi.nlm.nih.gov/pubmed/20613859)) to ensure that other relevant aspects of animal studies are adequately reported.

*2*

Human subjects

11. Identify the committee(s) approving the study protocol.

12. Include a statement confirming that informed consent was obtained from all subjects.

13. For publication of patient photos, include a statement confirming that consent to publish was obtained.

14. Report the clinical trial registration number (at [ClinicalTrials.gov](http://clinicaltrials.gov/) or equivalent).

Reported in section/paragraph or page #

| n/a |
| --- |
| n/a |
| n/a |
| n/a |

15. For phase II and III randomized controlled trials, please refer to the [CONSORT statement](http://www.consort-statement.org/) and submit the CONSORT checklist with your submission.

16. For tumor marker prognostic studies, we recommend that you follow the [REMARK reporting guidelines](http://www.nature.com/nrclinonc/journal/v2/n8/full/ncponc0252.html).

Data deposition

17. Provide accession codes for deposited data. Data deposition in a public repository is mandatory for:

a. Protein, DNA and RNA sequences

b. Macromolecular structures

c. Crystallographic data for small molecules

d. Microarray data

Reported in section/paragraph or page #

n/a

Deposition is strongly recommended for many other datasets for which structured public repositories exist; more details on our data policy are available in the Guide to Authors. We encourage the provision of other source data in supplementary information or in unstructured repositories such as [Figshare](http://www.figshare.com/) and [Dryad.](http://datadryad.org/) We encourage publication of Data Descriptors (see [Scientific](http://www.nature.com/sdata/) Data) to maximize data reuse.

18. If computer code was used to generate results

that are central to the paper’s conclusions,

include a statement in the Methods section

under “Code availability” to indicate whether n/a

and how the code can be accessed. Include

version information as necessary and any

restrictions on availability.

*3*
