# Supplementary material for: Activation of ALOX12 by a multi-organelle-orienting photosensitizer drives ACSL4-independent cell ferroptosis
Source: Cell Death Dis. 2022 Dec 14;13(12):1040. doi: 10.1038/s41419-022-05462-9 (PMC9751149; doi:10.1038/s41419-022-05462-9)
Supplement: Supplementary file 3 — Supplementary Information [file 41419_2022_5462_MOESM3_ESM.doc]

Supplementary Information

**Activation of ALOX12 by a multi-organelle-orienting photosensitizer drives ACSL4-independent cell ferroptosis**

*Xiuxia Wang,^1,#^ Yuanhong Chen,^2,#^ Xiang Yang,^2,#^ Lianghui Cheng,^2^ Zhenyan He,^2^ Yanru Xin,^2^ Shan Huang,^2^ Fanling Meng,^2^ Peijing Zhang,^2,3^,* Liang Luo^2,3,4^**

1. Henan Institute of Medical and Pharmaceutical Sciences, Zhengzhou University, Zhengzhou 450052, China
2. National Engineering Research Center for Nanomedicine, College of Life Science and Technology, Huazhong University of Science and Technology, Wuhan 430074, China
3. Key Laboratory of Molecular Biophysics of Ministry of Education, College of Life Science and Technology, Huazhong University of Science and Technology, Wuhan 430074, China
4. Hubei Key Laboratory of Bioinorganic Chemistry and Materia Medica, School of Chemistry and Chemical Engineering, Huazhong University of Science and Technology, Wuhan 430074, China

* Corresponding authors. Email: [zhangpeijing@hust.edu.cn](mailto:zhangpeijing@hust.edu.cn), [liangluo@hust.edu.cn](mailto:liangluo@hust.edu.cn)

^#^ These authors contributed equally to this work.

**1. Experimental Section**

**1.1 Materials**

3-(4,5-Dimethyl-2-Thiazolyl)-2,5-Diphenyl tetrazolium bromide (MTT), ciclopirox (CPX), methylene blue (MB), α-tocopherol (VE), fluoresceinc diacetate (FDA), propidium iodide (PI), phenylmethanesulfonyl fluoride (PMSF) and rosiglitazone (ROSI) were purchased from Energy Chemical (China). Mito-Tracker Deep Red FM, Lyso-Tracker Red DND-99, Endoplasmic Reticulum (ER)-Tracker Red, Golgi-Tracker Red (BODIPYTR® C5-Ceramide), RSL3 and BODIPY^581/591^ C11 were obtained from Thermo Fisher Scientific Company. Deferoxamine (DFO) was obtained from Sigma Aldrich Company (Shanghai, China). ML355 was obtained from Cayman Chemical. Reductive glutathione (GSH) and sodium ascorbate (VC) were purchased from Sinopharm (China). Necrostatin-1(Nec-1), 5-aminolevulinic acid hydrochloride (5-ALA), rose bengal (RB), zileuton, arachidonic acid (AA), doxorubicin hydrochloride (DOX), ferrostatin-1(Fer-1), and baicalein (BAI) were purchased from Aladdin. Dulbecco modified eagle medium (DMEM), fetal bovine serum (FBS), and penicillin-streptomycin mixture were provided by M&C Gene Technology Co., Ltd. (Beijing, China). N-phenylmaleimide (NP) was purchased from MACKLIN. Carbobenzyloxy-valyl-alanyl-aspartyl-[O-methyl]-fluoromethylketone (z-VAD-fmk) was purchased from Beyotime Biotechnology Company (Shanghai, China). TPCI, TPBT, TBCP, and BPCI were synthesized according to the literature[^1-3^](#_ENREF_1).

**1.2 Experimental Methods**

**Cell Culture**

HeLa, A498, BT549, H1299 and 786-O cancer cell lines were obtained from the American Type Culture Collection (ATCC). All cells were maintained in standard culture medium, which was DMEM supplemented with 10% (v/v) FBS, streptomycin (100 µg mL^-1^), and penicillin (100 U mL^−1^). All cells were cultured at 37 °C with 5% CO_2_.

**Cell Imaging**

For real-time imaging assay, HeLa cells (5 × 10^4^) were cultured in a chamber (LAB-NEST, Chambered Cover Glass System) for 24 h, and then cultured with TPCI solution (5 μM) in standard culture medium. The images of HeLa cells were taken by confocal laser scanning microscopy (CLSM, Olympus, FLUOVIEW FV3000, Japan) at 4 h post incubation with TPCI. The wavelength of excitation in CLSM was 488 nm (3% of power), and the emission signal was collected at 500–580 nm.

To examine the distribution of TPCI and TPCI@Lipo in cells, HeLa cells were pretreated with TPCI (1 μM) or TPCI@Lipo (TPCI, 1 μM)) for 24 h. The cells were washed by PBS twice before the solution of Mito-Tracker Deep Red FM (100 nM), ER-Tracker Red (1 μM), or Lyso-Tracker Red DND-99 (100 nM) was added and incubated for another 0.5 h. After that, the cells were washed by PBS for three times and imaged by CLSM. Cell staining by Golgi-Tracker Red was conducted according to the manual instruction post cells incubated with TPCI (1 μM) for 24 h. The wavelength of excitation in CLSM was 488 nm and signal collection was 500–580 nm for TPCI. The wavelength of excitation in CLSM was 594 nm and signal collection was 610–700 nm for Golgi-Tracker Red, Lyso-Tracker Red DND-99, and ER-Tracker Red. The wavelength of excitation in CLSM was 640 nm and signal collection was 650–750 nm for Mito-Tracker Deep Red FM.

**Different Photosensitizers Treatment**

Different types of human cancer cells (5 × 10^3^ cells per well) were seeded in a 96-well plate and cultured for 24 h. Subsequently, the cells were incubated with a photosensitizer (TPCI, TPBT, 5-ALA, or TPCI@Lipo in standard culture medium for 24 h; MB or RB in FBS-free DMEM medium for 1 h). The cells were washed and added with standard culture medium before light irradiation. Irradiation conditions were performed as following: 460 nm, 1 mW cm^-2^, 20 min for TPCI and TPBT; 640 nm, 5 mW cm^-2^, 20 min for MB; 520 nm, 5 mW cm^-2^, 5 min for RB; white light, 3.2 mW cm^-2^, 40 min for 5-ALA. For TPCI, TPBT, and RB, cell viability was determined using MTT assays at 4 h post irradiation. For MB and 5-ALA, cell viability was determined using MTT assays at 24 h post irradiation. The amount of various proteins and their messenger RNA levels in cells were measured at 24 h post irradiation. Unless indicated, the concentration of individual photosensitizer was used as following: 1 μM for TPCI, 2.8 μM for TPBT, 10 μM for MB, 5 μM for RB, 200 μM for 5-ALA, TPCI@Lipo (TPCI 1 μM).

**Western Blot (WB) Analysis**

After photosensitizers treatment, cell pellets were collected. The proteins were harvested from the cell pellets using lysis buffer (Beyotime, P0013, China) containing 1 mM PMSF, and their concentrations were determined by the BCA protein assay kit (Boster, AR1189, China). The proteins were separated by SDS-polyacrylamide gels (SDS-PAGE) and electrophoretically transferred onto polyvinylidene fluoride (PVDF) membranes (Biosharp, BS-PVDF-45, China). The membranes were blocked by 5% non-fat milk for 1–1.5 h at room temperature, and incubated with the corresponding primary antibodies at 4 °C overnight. The blots were incubated with horseradish peroxidase-conjugated secondary antibodies in 5% non-fat milk for 2 h at room temperature. The corresponding antigens were detected by ECL Kit (Boster, AR1111, China). Imaging was recorded using a ChemiDoc^TM^ XRS^+^ system (Biorad, USA) and quantified by Image Lab 5.2 software. GAPDH and β-actin were acted as internal control. Primary antibodies: GPX4 (Santa Cruz, sc-166570, WB: 1:1000), β-actin (ABclonal, AC026, WB: 1:5000), xCT/SLC7A11(D2M7A) rabbit mAb (CST, 12691S, WB: 1:1000), GAPDH (Santa Cruz, sc-47724, WB: 1:1000), anti-cleaved caspase-3 (Absin, abs132005, WB: 1:1000), Mouse anti-ACSL4(F-4) antibody (Santa Cruz, sc-365230, WB: 1:1000), Rabbit anti-ALOX15(C-term) polyclonal antibody (Absin, abs113269, WB: 1:1000), Mouse anti-ALOX12(C-5) (Santa Cruz, sc-365194, WB: 1:1000). Secondary antibodies: Horseradish peroxidase-conjugated anti-mouse (Absin, abs20001, WB: 1:10000) and anti-rabbit (ABP Biosciences, L153, WB: 1:10000) secondary antibody.

**MTT Assays**

After photosensitizers treatments, the cells were incubated with fresh MTT (0.5 mg mL^-1^) solution for 4 h. The medium was replaced with 150 µL of DMSO to dissolve formanzan. The absorbance at 570 nm was measured with a microplate reader (Thermo Scientific, Varioskan LUX, USA) and the cell viability was calculated as (OD_570_ sample - OD_570_ blank)/(OD_570_ control- OD_570_ blank ) × 100%, where OD_570_ control was obtained in the absence of agents, OD_570_ blank was the plate absorption, and OD_570_ sample was obtained after co-incubation with the mixtures. Each test group contained at least three replicates and each experiment was repeated at least three times.

**FDA/PI Staining**

FDA/PI staining was used to visualize the number of living and dead cells. Specifically, HeLa cells (5 × 10^4^ cells per well) were seeded into 12-well plates and incubated for 24 h, followed by incubation with TPCI (1 μM) for 24 h. For the non-irradiation group (“Dark” group), the cells were kept in dark before staining. For the irradiation group, the cells were exposed to the irradiation (460 nm, 1 mW cm^-2^, 20 min). The cells in both groups were stained by a mixed solution of FDA (10 μg mL^-1^) and PI (20 μg mL^-1^). After incubation for another 10 min and being washed with PBS, the cells were imaged by a fluorescence microscope (Micro-shot Optical Technology Co., Ltd., MF52, China).

**Inhibitors Assays**

Various cancer cells were seeded in 96-well plates. After 24 h of incubation, the cells were exposed to different treatments as following:

1. The photosensitizer and one of the following inhibitors were added into the cells and co-incubated for 24 h before irradiation: apoptosis inhibitor (z-VAD-fmk, 50 µM), necroptosis inhibitor (Nec-1, 50 µM), reactive oxygen species eliminators (VC, 400 µM; VE 200 µM), apoptosis inducing factor inhibitor (N-phenylmaleimide, NP, 10 µM), ACSL4 inhibitor (ROSI, 20 µM or 40 µM), or LOX inhibitors (baicalein, zileuton, ML355).
2. After incubating the cells with the photosensitizer for 22 h, one of the following inhibitors was added and co-incubated with the cells for another 2 h before irradiation: ferroptosis inhibitor (Fer-1, 50 µM or 100 µM), reductive GSH, DFO (20 µM), or CPX (4 µM).

After irradiation, the cell viability was measured using MTT assays at 4 h (TPCI, TPBT, and RB) or 24 h (MB and 5-ALA) post irradiation. The rescue rate was calculated to indicate the protective effects of each inhibitor, which was compiled in a heat map drawn by Prism 8 (Graphpad Software, LLC). Rescue rate was determined by the following formula:

Rescue rate = Cell viability in a photosensitizer treatment with an inhibitor- Cell viability in a photosensitizer treatment without an inhibitor

**Detection of Cell Membranes Permeability**

At 1 h post irradiation, the HeLa cells treated with TPCI (1 µM) were added with a PI solution (40 μg mL^-1^) solution and incubated for 15 min. Fluorescence images of cells were taken by the fluorescence microscope.

**Transmission Electron Microscopy (TEM)**

Samples for TEM imaging were fixed with 2.5% glutaraldehyde in PBS solution at 4 °C for 24 h. The samples were stained with 4% osmium tetroxide for 0.5 h at room temperature, and washed by PBS for 3 times. The samples were then dehydrated by a series of ethanol/water mixed solutions (ethanol fraction 30%, 50%, 70%, 80%, 85%, and 90%, respectively), and rinsed by 100% ethanol twice. The samples were embedded in epoxy resin and stored at 60 °C for 48 h until the resin polymerized after permeation. Afterwards, the samples were sliced to thickness of 80–100 nm, and stained with 5% uranyl acetate for 15 min and 2% lead citrate for 15 min before imaging, TEM images were taken by a transmission electron microscope (FEI Company, Tecnai G2 TWIN, USA).

**Measurement of Mitochondrial Membrane Potential**

HeLa cells (2 × 10^5^ cells per well) were seeded in a 6-well plate and cultured for 24 h. Subsequently, the cells were incubated with TPCI (1 μM) for 24 h or DOX (20 μM) for 5 h. For the non-irradiation group (“Dark” group), the cells were kept in dark before staining. For the irradiation group, the cells were washed and added with standard culture medium before light irradiation (460 nm, 1 mW cm^-2^, 20 min). After irradiation, the HeLa cells were immediately trypsinized and stained by Mito-Tracker Deep Red FM (100 nM) for 20 min. Labeled cells were resuspended in PBS and analyzed using a flow cytometer (Beckman, CytoFLEX S, USA).

**Lipid ROS-Generation Measured by BODIPY^581/591^ C11**

The generation of lipid ROS were measured following the literature procedures[^4-7^](#_ENREF_4). After the addition of TPCI (1 μM), ferroptosis inhibitors (Fer-1, 50 µM) or VE (200 µM) were added into each well immediately and co-incubated with cells for 24 h. The cells were then washed by PBS and stained by BODIPY^581/591^ C11 (5 μM) for 30 min before being exposed to light irradiation (460 nm, 1 mW cm^-2^, 20 min). The cells were imaged by CLSM and fluorescence microscope, and the fluorescence intensity of BODIPY^581/591^ C11 (*λ*_ex_ = 575 nm, *λ*_em_ = 600 nm) was measured by a microplate reader.

**Measurement of Intracellular Malondialdehyde (MDA)**

The intracellular lipid ROS were measured by the thiobarbituric acid reaction. At 1 h after TPCI treatment, HeLa cells were lysed by the lysis buffer (Beyotime, P0013, China) on ice. The lysates were centrifuged at 10,000 rpm for 10 min at 4 °C. The supernatant was collected and the total proteins were determined by a BCA protein assay kit. The MDA assay was performed in a lipid peroxidation MDA assay kit (Beyotime, S0131S, China). Thiobarbituric acid reactive substances (MDA-TBA) values were calculated as nM/mg of the total protein. The relative level was normalized to the MDA content in pristine HeLa cells.

To perform MDA measurement in tumor tissues, each tumor tissue was mixed with 500 μL RIPA lysis buffer (Beyotime, P0013, China) and homogenized on ice. The samples were centrifuged at 10,000 rpm for 10 min at 4 °C, and the supernatant was collected and analyzed by the MDA assay.

**Real-Time qPCR**

At the end of the treatment, HeLa cells or mice tumor tissues were homogenized with TRIzol reagent (Vazyme Biotech Co., Ltd., R401-01, China). Total RNA was isolated following the manufacturer’s protocol, and 1 µg of total RNA was reverse transcribed using HiScript® II Q RT SuperMix for qPCR (Vazyme Biotech Co., Ltd, R223-01, China). Different gene expression was quantified with SYBR® Green Real time PCR Master Mix (Vazyme Biotech Co., Ltd, Q311-02, China). Reactions were carried out on the Bio-Rad CFX connected real-time system (Bio-Rad, USA), and the threshold cycle values were calculated by Bio-Rad CFX maestro software. Expression levels of the genes were normalized to the housekeeping gene *GAPDH*, and the induced fold over control was calculated using the 2^-ΔΔCt^ methods.

**Table 1** Primers for RT-PCR

| **Genes** | **Sequences** |
| --- | --- |
| GAPDH | F: TCCCTGAGCTGAACGGGAAG; R: GGAGGAGTGGGTGTCGCTGT |
| ALOX12 | F: TTCAAATGGCCATCTCATGGCATCTGAGT;  R: ATCTGTTCGGAATTGGTTTAGCACAGCTTT |
| p53 | F: CTTTGAGGTGCGTGTTTGTGC; R: AGTGCTCGCTTAGTGCTCCCT |
| SLC7A11 | F: GGCAGTTGCTGGGCTGATTTA; R: GATGACGAAGCCAATCCCTGT |
| PTGS2 | F: CTCAGCCATACAGCAAATCCTT; R:GTCCGGGTACAATCGCACTTAT |
| LPCAT3 | F: CATCTCCATCTTCCTGGGTTACC; R: GTGCGGCCCATTAGTCGAA |
| GRP78 | F: ACCGCTGAGGCTTATTTGGG; R: CTGCCGTAGGCTCGTTGAT |
| GM130 | F: CCCAAGGACAATGCTGCTACTC; R: CGCAGACTCACAAACAAGACCAT |
| CHOP | F: ACCAAGGGAGAACCAGGAAACG; R: TCACCATTCGGTCAATCAGAGC |

**Intracellular GSH Content**

HeLa cells seeded in a cell-culture dish were cultured for 24 h. At 1 h post TPCI treatment, HeLa cells lysates were collected. Reductive GSH and oxidized glutathione (GSSG) were evaluated with a GSH/GSSG qualification kit (Beyotime, S0053, China).

**Generating Stable Cell Lines**

Lenti-myc-GPX4, lenti-myc-ALOX15, and lenti-Flag-ALOX12-cherry recombinant plasmids were generated by cloning the full length open reading frame (ORF) of human gene GPX4 (NM_002085.5), ALOX15 (NM_001140.5), and ALOX12 (NM_000697.3) fused with cherry into lentiviral vector pLenti CMV Puro DEST (w118-1) (Addgene) using Gateway recombinant cloning technology (Thermofisher). The scramble shRNA, GPX4 shRNA, ALOX12 shRNA, and ALOX15 shRNA were generated by ligating the oligonucleotides (**see Table 2**) into pLKO.1-TRC according to the protocol from Addgene. pCDH-Flag-ALOX12-EF1a-blasticidin (pCDH-Flag-ALOX12) recombinant plasmid was established by cloning the full length ORF of human gene ALOX12 into the lentiviral vector pCDH-CMV-MCS-EF1a-blastcidin using Gibson assembly cloning technology (Beyotime, Jiangsu, China). In this study, pCDH-Flag-ALOX12 was employed to rescue the ALOX12 knockdown in HeLa cells. In order to impair the effect of ALOX12 shRNA to exogenous ALOX12, six amino acid synonymous mutations were generated at the binding sites of ALOX12 shRNA in pCDH-Flag-ALOX12 using the Hieff Mut™ Multi Site-Directed Mutagenesis Kit (Yeasen Biotechnology Co., Ltd., Shanghai, China), the specific primers for mutation in ALOX12 were listed in **Table 2**. The lentiviral vectors were co-transfected with packaging vectors psPAX2 and pMD2G (Addgene) into 293T cells for lentivirus production. To establish stable cell lines, cancer cells (HeLa, H1299, 786-O) were infected using the above lentivirus. After 48 h of infection, positive cells were selected with 1 μg mL^-1^ puromycin or 20 μg mL^-1^ blasticidin for 4 days.

**shRNA-Mediated Genes Knockdown in Cancer Cells**

We inserted the annealed oligonucleotides (see Table 2) between the AgeI and EcoRI sites of pLKO.1-TRC according to the protocol from Addgene. Lentivirus production, lentivirus transfection, and stable cell lines construction were performed as described above in “**Generating Stable Cell Lines**”. Genes knockdown were ensured with western blot analysis and/ or RT-qPCR analysis.

**Table 2** The oligonucleotides list was used to generate shRNA

| **shRNA** | **oligonucleotides synthesis** | |
| --- | --- | --- |
| Scramble-shRNA | | CCGGCAACAAGATGAAGAGCACCAACTCGAGTTGGTGCTCTTCATCTTGTTGTTTTTG |
| GPX4-shRNA | | CCGGGTGAGGCAAGACCGAAGTAAACTCGAGTTTACTTCGGTCTTGCCTCACTTTTTG |
| ALOX12-shRNA#1 | | CCGGGCATCGAGAGAAGGAACTGAACTCGAGTTCAGTTCCTTCTCTCGATGCTTTTTG |
| ALOX12-shRNA#2 | | CCGGCCAAAGGGATGACATAGTGAACTCGAGTTCACTATGTCATCCCTTTGGTTTTTG |
| ALOX15-shRNA | | AATTCAAAAAGCTATCAAAGACTCTCTAAATCTCGAGATTTAGAGAGTCTTTGATAGC |
| Mutant-ALOX12 | | GCACCGTGAAAAAGAGCTAAAAGACAGACAGCAGATCT |

**siRNA-Mediated Knockdown of ACSL4**

HeLa cells were seeded into 24-well plate and transfected with 0.2 nmoL siRNA (**see Table 3**) and Lipofectamine 2000 (Invitrogen). Transfection was performed for 6 h at 37 °C. Two days the transfection step, the ACSL4 expression was detected by western blot. And then the sensitivity of ACSL4-knockdown HeLa cells to TPCI treatment was evaluated by MTT assays.

**Table 3** The oligonucleotides list of siRNAs

| **siRNA** | **oligonucleotides synthesis** | |
| --- | --- | --- |
| Negative control | | UUCUCCGAACGUGUCACGUTT |
| siACSL4-#1 | | GCAGAUACUCUGGAUAAAUTT |
| siACSL4-#2 | | GCAGUAGUUCAUGGGCUAATT |
| siACSL4-#3 | | CCAAGUAGACCAACGCCUUTT |

**CRISPR-Cas9–mediated knockout of ALOX12 and ACLS4**

To knockout ALOX12 and ACLS4 in HeLa cells, lentiCRISPR v2 plasmid (encoding guide RNA, the endonuclease Cas9) with guide RNA specific to GFP, ALOX12 and ACLS4 was constructed according to the protocol from Addgene. Here the guide GFP was used as the control. After co-transfected with packaging vectors psPAX2 and pMD2G (Addgene) into 293T cells for lentivirus production. HeLa cells were infected using the above lentivirus. After 48 h of infection, positive cells were selected with puromycin (1 μg mL^-1^) for 7 days, and clones propagated from single cells were picked out. The depletion of ALOX12 and ACSL4 was confirmed by both Western blot analysis (antibodies identified above) and DNA sequencing. The target sequences for guide RNA were as follows:

GFP guide RNA: 5′-GGGCGAGGAGCTGTTCACCG-3′,

ALOX12 guide RNA 1: 5′-GTTTGATCATGACGTTGCAG -3′,

ALOX12 guide RNA 2: 5′- GGTCCGGCCTGCACAGGAGG-3′,

ACSL4 guide RNA 1: 5′-TGCAATCATCCATTCGGCCC-3′,

ACSL4 guide RNA 2: 5′- TCCCAAACTTGGATACAGCA -3′.

**Purification of ALOX12 in 293 T Cells**

The full-length ORF of human gene ALOX12 (NM_000697.3) was amplified by PCR. The coding sequence of ALOX12 fused with N-terminal SFB tag was inserted into the lentiviral vector pLenti CMV Puro DEST (w 118-1) (Addgene) using Gibson assembly cloning technology (Beyotime, Jiangsu, China). The recombinant plasmids were transfected into 293T cells for 48 h, the cells were harvested and lysed by sonication in PBS solution supplemented with protease inhibitor cocktail (Boster, Wuhan, China). After centrifugation, the supernatant was transferred into a clean new tube and incubated with 100 μL strepavidin agarose beads (GE Health, USA) overnight at 4 °C. The beads were washed with PBST buffer (PBS supplemented with 0.5% Tween 20) and the SFB-ALOX12 protein was eluted with elution buffer (PBS supplemented with 1.5 mg mL^-1^ biotin). The eluted proteins were further desalted and enriched using Millipore Ultra-filtration column (MWCO 10 KD). SFB-ALOX12 proteins were pooled, and stored at -80 °C prior to the enzymatic assay. The purity of SFB-ALOX12 proteins was more than 90%, which was determined by SDS-PAGE analysis.

**Activity Assay of ALOX12 *in vitro***

SFB-ALOX12 enzymatic activity were determined by detecting the formation of the peroxidation product of AA, 12(*S*)-hydroperoxy tetraenoic eicosatetraenoic acid (12(*S*)-HpETE) (Extinction coefficient = 27,000 M^-1^ cm^-1^), at 234 nm using a UV/Vis spectrophotometer (Puxi General Instrumental Company, TU-1810DSPC, China). The reaction medium was PBS containing 0.2 mM ATP, 0.01% Triton X-100, and 0.3 mM Ca^2+^ (pH = 8.00). The reactions started by adding approximately 3.6 μg enzyme to a 0.5 mL reaction medium containing 100 µM AA, with or without ML-355 (10 µM) or TPCI (5  µM), at 25 °C, along with constant shaking. For the “TPCI” group, enzyme ALOX12 was added into the reaction medium containing 5 µM TPCI and exposed to irradiation (460 nm, 35 mW cm^-2^, 15 min), and AA was added into the mixture next. After 30 min, 20 µL of the reaction mixture was taken and dissolved in 2 mL ethanol, before being submitted to UV/Vis measurement. To study whether ALOX12 was necessary on the production of 12(*S*)-HpETE, a reaction mixture containing 5 µM TPCI but without ALOX12 was exposed to the same irradiation, followed by adding AA into the mixture. After 15 min, 20 µL of reaction mixture was taken and dissolved in 2 mL ethanol before UV/Vis absorption measurement. The test was repeated for triplicate times.

**Computational Modeling**

The molecular docking modeling was employed to study the interactions between various photosensitizers and human ALOX12 (Uniprot ID P18054). All photosensitizers were built with ChemDraw software and optimized to obtain stable formation conformations. The photosensitizers were docked to the protein using AutoDock, version 4.2.0 (<http://www.scripps.edu/mb/olson/doc/autodock>) and AutoDock Vina, based on Lamarckian Genetic Algorithm (LGA)[^8^](#_ENREF_8). The photosensitizer was set as a ligand and ALOX12 was set as a receptor. We used a large grid box with dimensions of 126 × 126 × 126 Å for the docking modeling, due to the large size of ALOX12 protein. The best model was selected among different models, in which the photosensitizer bound to the catalytic site of ALOX12 with the highest binding affinity (the lowest binding energy). **Preparation and Characterization of Liposomes**

The preparation of liposomes loaded with TPCI (named as TPCI@Lipo) was following literature procedures[^9^](#_ENREF_9). The size (diameter, nm), and size distribution (PDI) of TPCI@Lipo were measured by dynamic light scattering (Malvern Zetasizer, Nano ZS90, USA). The concentration of TPCI in TPCI@Lipo was calculated based on the absorbance at 442 nm.

**Pearson Correlation Coefficient Calculation**

The background of eight-bit greyscale TIFF images were deducted based on their minimal pixel intensity values. The integrated pixel intensities of TPCI (green signals) as well as mitochondria, lysosomes, ER, Golgi apparatus (red signals) were conducted by Image J software. A Pearson correlation coefficient (*P*) between the green signals and the red signals was calculated according to previous literatures[^10^](#_ENREF_10).

***In Vivo* Antitumor Efficacy**

Female BALB/c nude mice (four-weeks-old) were purchased from HuaFuKang Laboratory Animal Centre of Beijing, China. HeLa cancer cells (5 × 10^6^) were subcutaneously injected into the right rear flank of each mouse to establish HeLa tumor-bearing mouse model. All the animal experiments were approved by the Institutional Animal Care and Ethic Committee of Huazhong University of Science and Technology.

When the tumor size reached approximately 100 mm^3^, the mice were randomly divided into five groups (9-10 mice for each). The mice in the “Control” group were dosed with saline (25 µL, 0.9% NaCl) on Day 0 and Day 2. The mice in the “TPCI-PDT” group were dosed with TPCI (25 µL, 2.4 mg mL^-1^) on Day 0 and Day 2, and subjected to irradiation (460 nm, 200 mW cm^-2^, 6 min) daily on Day 0 to Day 3. The mice in the “TPCI-PDT + ML355” group were dosed with TPCI (25 µL, 2.4 mg mL^-1^) on Day 0 and Day 2 and subjected to irradiation (460 nm, 200 mW cm^-2^) for 6 min daily on Day 0 to Day 3. The mice in this group were also dosed with ML355 (25 µL, 2.4 mg mL^-1^) daily from Day 0 to Day 3. The mice in the “DOX” group were dosed with DOX (25 µL, 2.4 mg mL^-1^). All mice were dosed intratumorally. For the groups without irradiation, mice were kept in the dark after administration. After various treatments, the tumor sizes were measured every day by a Vernier caliper, and calculated by the equation V = L × W^2^ / 2 (L and W were the longer diameter and shorter diameter of the tumor, respectively).

**Histological Examination**

On Day 2, four mice in each group were sacrificed and the tumor tissue on each mouse was dissected. The tumor tissues were kept in 4% paraformaldehyde for further hematoxylin and eosin (H&E) staining, Ki67 staining, and cleaved caspase-3 staining assays. For H&E staining, the tumor sections were stained according to the standard protocols[^11^](#_ENREF_11). For Ki67 staining, the tumor sections were stained according to the instruction manual of Ki67 detection kit (Servicebio, GB111499). For cleaved caspase-3 staining, the tumor slides were permeabilized with PBS containing 0.2% Txiton X-100 before incubation with 10% goat serum (Servicebio, WGAR1009-5) for 30 min at room temperature. The sections were incubated with anti-cleaved caspase-3 (Servicebio, GB13436, 1:100 dilution) overnight at 4 °C in a humidified chamber. Sections were washed by PBST for three times before incubating with goat anti-rabbit IgG secondary antibody (Servicebio, GB1213). The images of tumor tissue slides were measured by an optical microscope (Zeiss, Germany).

**Statistical Analysis**

Statistical analysis was performed using GraphPad Prism 8 to assess the differences between experimental groups. All quantitative results were expressed as mean ± standard deviation (SD). Statistical significance was analyzed using a two-tailed, unpaired Student’s t-test with a confidence interval of 95%. *P* values of < 0.05 and < 0.01 were considered statistical difference and statistically significant difference, respectively.

**2. Figures**


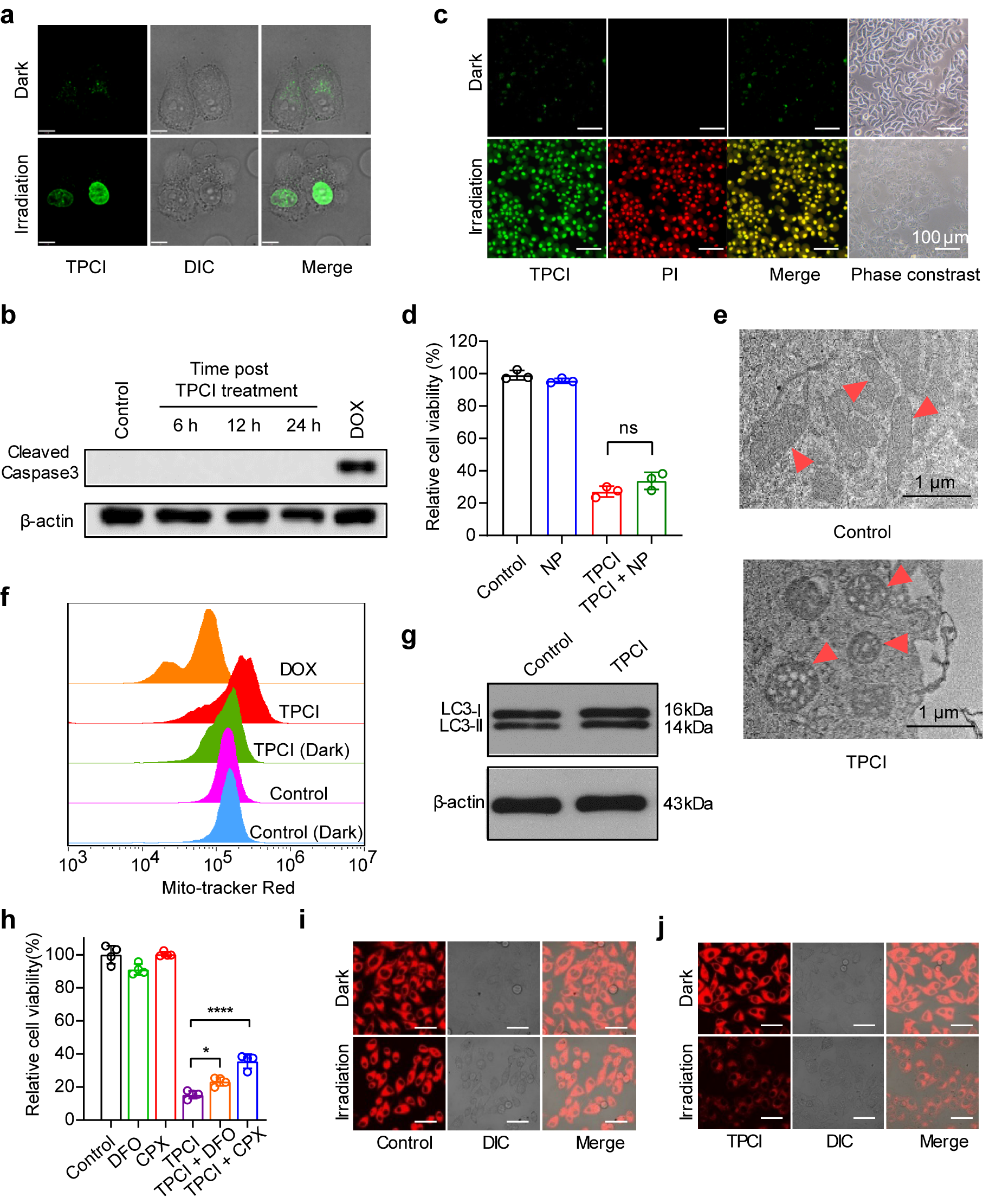


**Supplementary Fig. 1.** **a**, The CLSM images of HeLa cells pretreated with TPCI (5 μM) with and without irradiation (488 nm laser from CLSM, 3% of power, 1 min). **b,** Western blot of cleaved caspase-3 and β-actin expression in HeLa cells at different time after TPCI treatment. TPCI concentration 1 μM. DOX (2 μM) treated with HeLa cells for 24 h was used as a positive control. The experiments were repeated three times independently with similar results. **c**, Fluorescence images of HeLa cells treated with TPCI (1 μM) and PI (40 μg mL^-1^) either in dark or upon irradiation (460 nm, 1 mW cm^-2^, 20 min). scale bar: 100 μm. **d**, Relative viability of HeLa cells receiving TPCI treatment, treated with or without N-phenylmaleimide (NP, 10 μM). **e**, Transmission electron microscopy (TEM) of HeLa cells in TPCI treatment. The red arrows referred to mitochondria. **f**, Fluorescence of Mito-Tracker Deep Red in HeLa cells treated with DOX (20 μM) or TPCI treatment. **g**, LC3 expression in HeLa cells with and without TPCI treatment. **h**, Relative viability of HeLa cells receiving TPCI treatment, treated with or without deferoxamine (DFO, 20 μM), or ciclopirox olamine (CPX, 4 μM). TPCI concentration: 1 μM; Irradiation conditions: 460 nm, 1 mW cm^-2^, 20 min. **i**, The CLSM images of HeLa cells incubated with BODIPY581/591 C11 (5 μM) with or without irradiation (460 nm, 1 mW cm^-2^, 10 min). Scale bar: 50 μm. **j**, The CLSM images of HeLa cells co-incubated with TPCI (1 μM) and BODIPY581/591 C11 (5 μM) with or without irradiation (460 nm, 1 mW cm^-2^, 10 min). λex = 594 nm, λem = 610–700 nm, scale bar: 50 μm. TPCI concentration: 1 μM; Irradiation conditions: 460 nm, 1 mW cm^-2^, 20 min. Data were shown as mean ± SD from a representative experiment (n = 3–4) of 2–3 independent biological replicates. Statistical significance was determined by two-tailed unpaired Student's t-test (ns: no significance, **P* < 0.05, and *****P* < 0.0001).


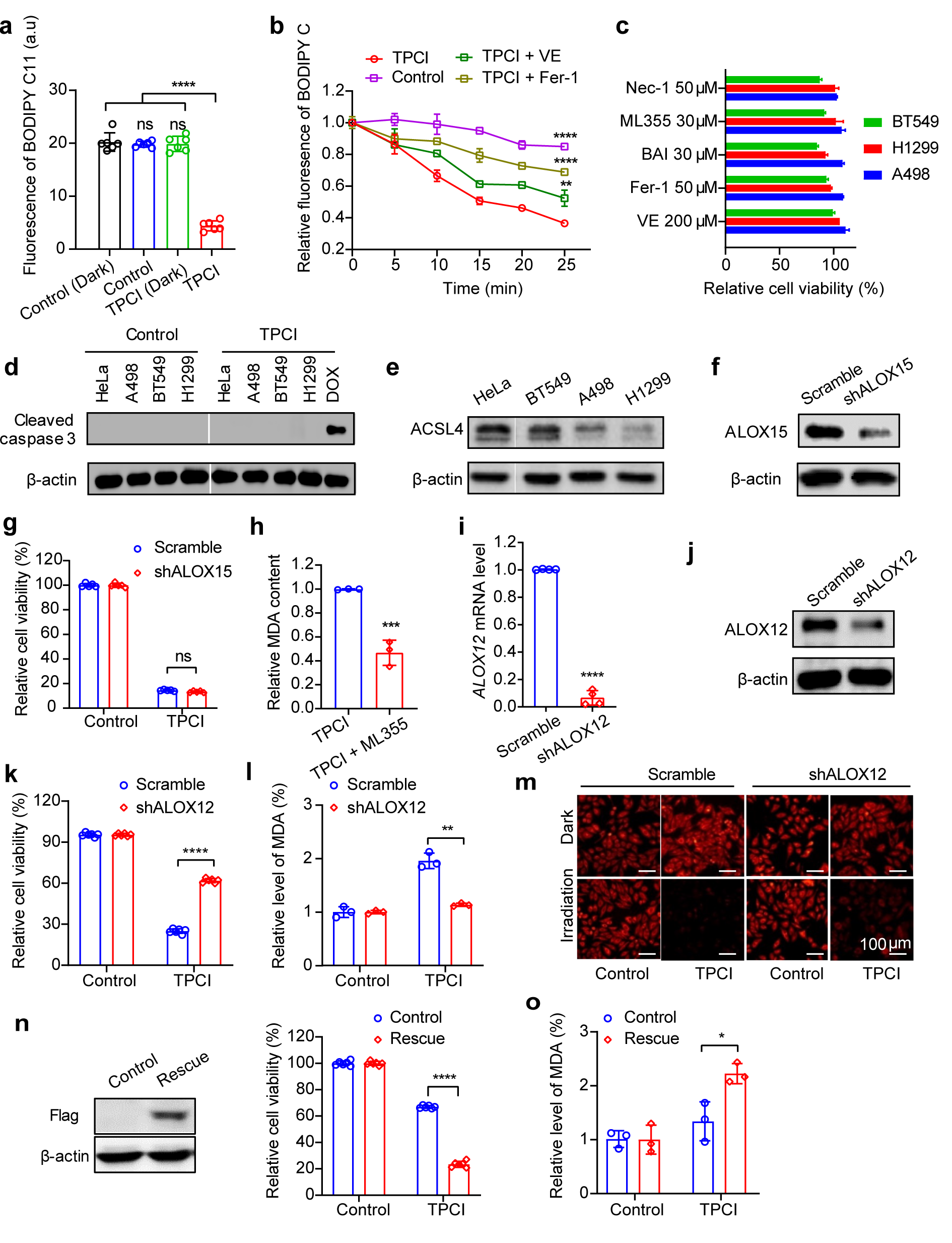


**Supplementary Fig. 2. a**, The intrinsic red fluorescence signal of BODIPY^581/591^ C11 in HeLa cells receiving TPCI treatment. TPCI concentration: 1 μM; Irradiation conditions: 460 nm, 1 mW cm^-2^, 20 min. **b**, The relative red fluorescence signal of BODIPY^581/591^ C11 in HeLa cells receiving TPCI treatment, treated with VE (200 μM) or Fer-1 (50 μM), as a function of irradiation time. The density of blue light power was 1 mW cm^-2^. **c**, Relative viability of various cancer cell lines treated with different inhibitors. **d**, Immunoblot analysis of cleaved caspase-3 in various cancer cells exposed to TPCI treatment. **e**, Immunoblot analysis of ACSL4 expression in various cancer cells. The data were shown as mean ± SD from a representative experiment (n = 4) from of 2 independent biological replicates. **f**, Knockdown of ALOX15 by shRNAs (shALOX15) in HeLa cells, determined by western blotting of ALOX15. **g**, Relative viability of the scramble and ALOX15-knockdown HeLa cells receiving TPCI treatment. **h**, The MDA contents of HeLa cells receiving TPCI treatment, treated with or without ML355 (30 μM). **i**, *ALOX12* mRNA expression of the scramble and ALOX12-knockdown (shALOX12) HeLa cells. **j**, Knockdown of ALOX12 expression by shRNA in HeLa cells determined by western blotting of ALOX12. **k**, Relative viability of the scramble and the ALOX12-knockdown HeLa cells treated by TPCI and light irradiation. **l**, Relative MDA levels in the scramble and the ALOX12-knockdown HeLa cells treated by TPCI and light irradiation. **m**, Fluorescence images of different HeLa cells (scramble and ALOX12-knockdown HeLa cells) receiving TPCI treatment, stained with BODIPY^581/591^ C11 (5 μM). **n**, Left: Recovery of ALOX12 expression in HeLa cells determined by western blotting of Flag. Right: Cell viability of the control and the ALOX12-recovered HeLa cells treated by TPCI and light irradiation. **o**, Relative MDA level in the control and the ALOX12 rescued HeLa cells. TPCI concentration: 1 μM; Irradiation conditions: 460nm, 1 mW cm^-2^, 20 min. Data were shown as mean ± SD from a representative experiment (n = 3–6) of 2–3 independent biological replicates. Statistical significance was determined by two-tailed unpaired Student's t-test (ns: no significance, **P* < 0.05, ***P* < 0.01, ****P* < 0.001, and *****P* < 0.0001).


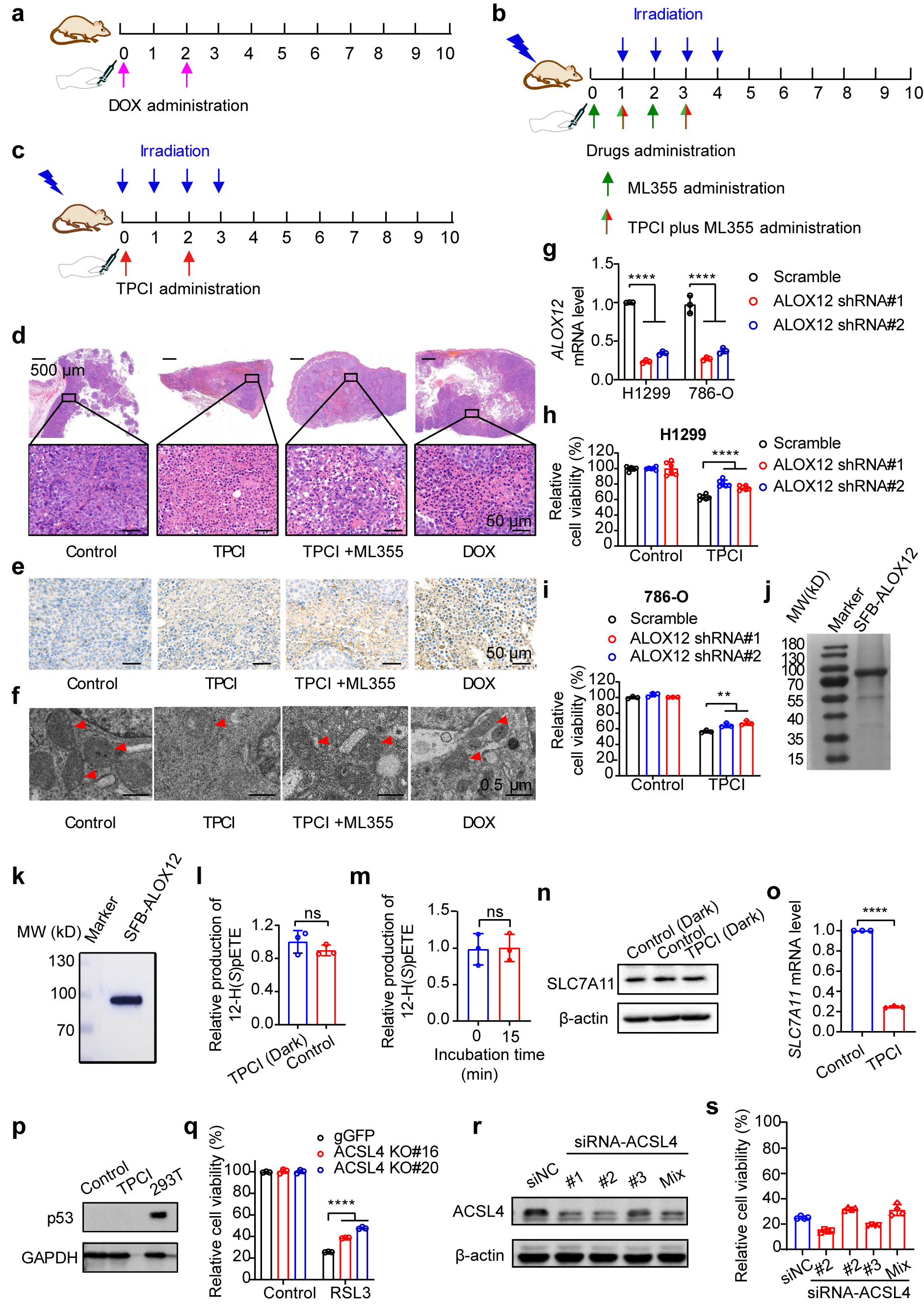


**Supplementary Fig. 3.** The time axis of the *in vivo* study of HeLa tumor-bearing nude mice model when the tumor volume reached about 100 mm^3^. **a**, “DOX” group; **b**, “TPCI” group; **c**, “TPCI + ML355” group. **d**-**e**, Immunohistochemical analysis of the dissected tumors on Day 2 post treatment to reveal necrosis using H&E staining (**d**), and caspase-3 activation (**e**) using anti-cleaved caspase-3 antibody. **f**, Representative TEM images of dissected tumors on Day 2 post treatment from various groups. The red arrows referred to mitochondria. **g**, RT-PCR analysis of mRNA expression of ALOX12 in indicated cells. **h-i**, Relative viability of the scramble and the ALOX12-knockdown H1299 (h) or 786-O cells (i) treated by TPCI and light irradiation. **j**, The SDS-PAGE of SFB-ALOX12. **k**, The SFB-ALOX12 determined by western blotting of ALOX12. **l**, The relative production of 12-H(*S*)pETE of AA (100 μM) in the presence of TPCI (5 μM) without irradiation. **m**, The relative production of 12-H(*S*)pETE of AA (100 μM) in the presence of TPCI (5 μM) with irradiation (460nm, 5 mW cm^-2^, 3.5 min). **n**, The SLC7A11 expression of HeLa cells treated with TPCI (1 μM) without light irradiation. **o**, mRNA expression levels of *SLC7A11* gene from HeLa cells receiving TPCI treatment. **p**, p53 expression of HeLa cells (control) and HeLa cells receiving TPCI treatment. p53 expression in 293T cells without any treatment was detected to ensure the [quality](javascript:;) of p53 primary antibody. **q**, Relative viability of the control CRISPR (gGFP) or ACSL4-knockout (KO) HeLa cells treated by RSL3 (7.5 μM, 24 h). **r**, Western blot analysis of HeLa cells transfected with the control siRNA (siNC) and different specific ACSL4 siRNAs. **s**, Relative cell viability of the siNC and the ACSL4-knockdown HeLa cells treated by TPCI and light irradiation. TPCI concentration was 1 μM; Irradiation: 460nm, 1 mW cm^-2^, 20 min. The data were shown as mean ± SD from a representative experiment (n = 3) of 2–3 independent biological replicates. Statistical significance was analyzed by two-tailed unpaired Student's t-test (ns: no significance, ***P* < 0.01, and *****P* < 0.0001).


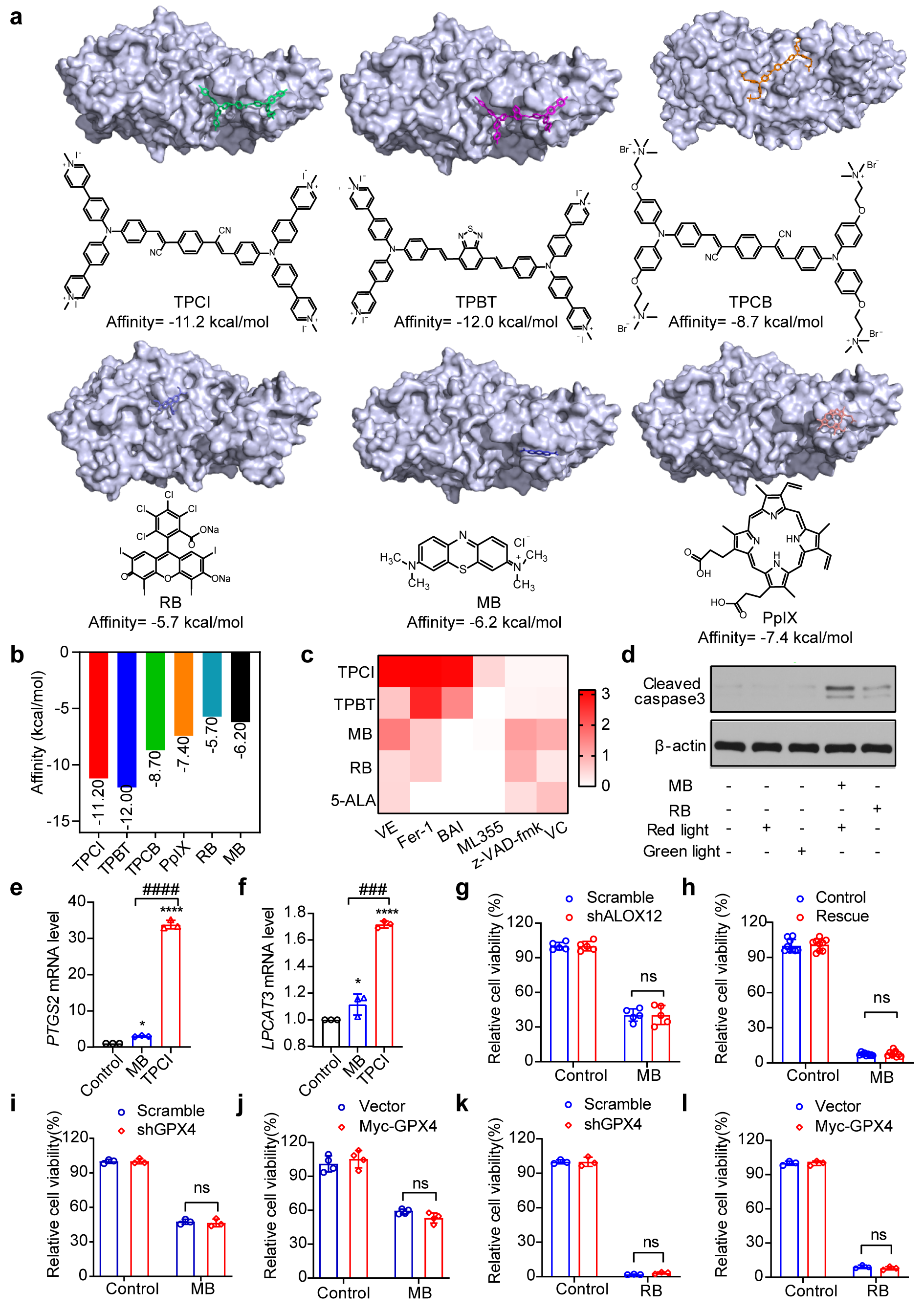


**Supplementary Fig. 4. a**, Molecular docking modeling between ALOX12 and various photosensitizers. **b**, The affinity of ALOX12 to different photosensitizers. **c**, Hot map of HeLa cells rescued by various inhibitors after receiving different photosensitizers treatment. **d**, Western blotting analysis of cleaved caspase-3 in HeLa cells with MB or RB treatment. **e**-**f**, Relative mRNA level of *PTGS2* gene (**e**), and *LPCAT3* gene (**f**) in HeLa cells with MB or RB treatment. Data difference shown were compared to the “Control” group. **g**, Relative viability of the scramble and ALOX12-knockdown HeLa cells with MB treatment. **h**, The influence of the recovered ALOX12 expression in viability of HeLa cells with MB treatment. **i**, Relative viability of the scramble and GPX4-knockdown HeLa cells with MB treatment. **j**, Relative viability of the vector and GPX4-overexpressed HeLa cells with MB treatment. **k**, Relative viability of the scramble and GPX4-knockdown HeLa cells with RB treatment. **l**, Relative viability of the vector and GPX4-overexpressed HeLa cells with RB treatment. Different photosensitizers treatment: concentrations of MB, RB, and 5-ALA in PDT were 10 μM, 5 μM, and 200 μM, respectively. Irradiation for MB treatment: 640 nm, 5 mW cm^-2^, 20 min; RB treatment: 520 nm, 5 mW cm^-2^, 5 min; 5-ALA treatment: white light, 3.2 mW cm^-2^, 40 min. The data were shown as mean ± SD from a representative experiment (n = 3–9) of 2–3 independent biological replicates. Statistical significance was analyzed by using two-tailed unpaired Student's t-test (ns: no significance, **P* < 0.05, and *****P* < 0.0001).


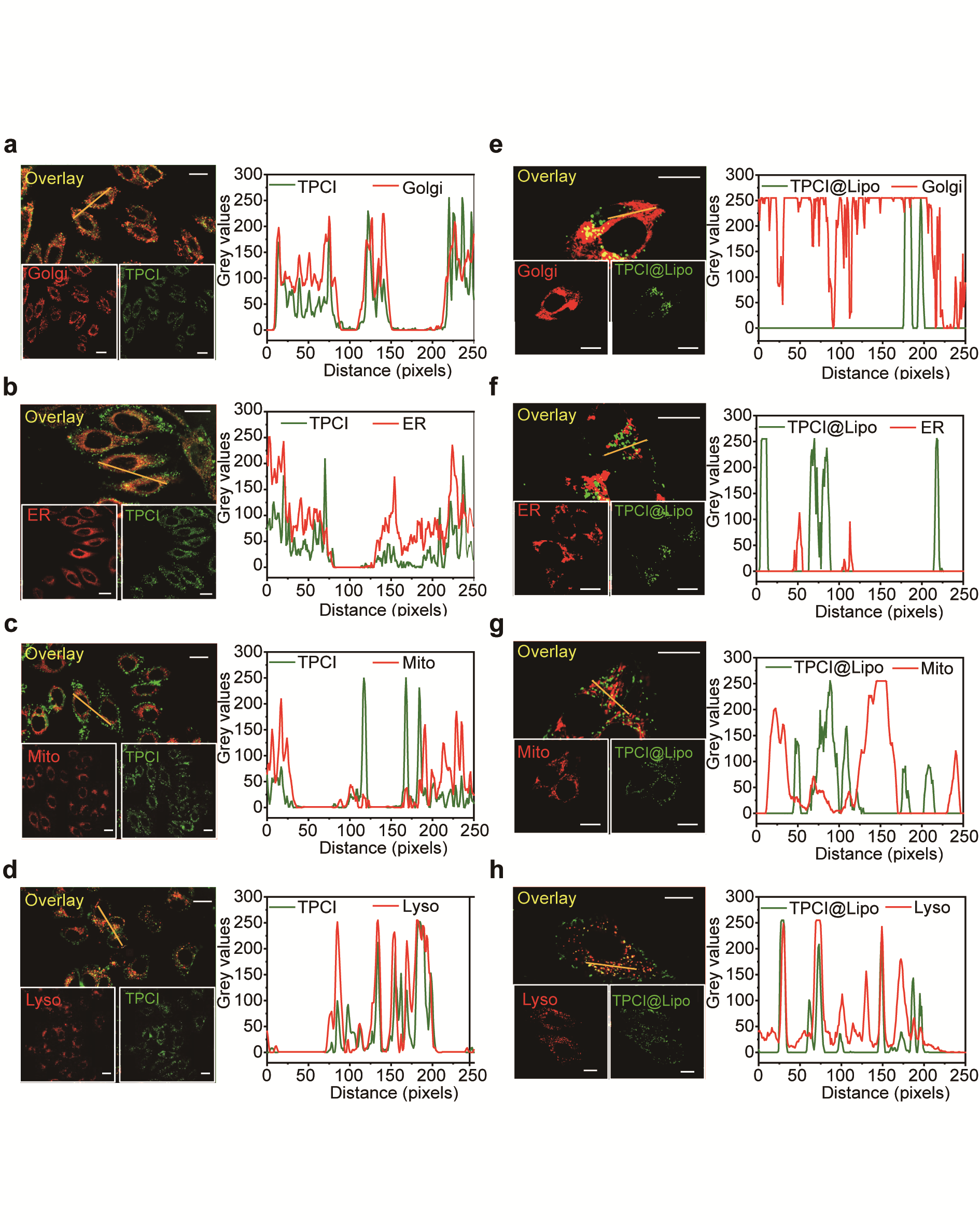


**Supplementary Fig. 5.** **a**–**d**, Colocalization of TPCI and Golgi-Tracker Red (**a**), ER-Tracker Red (**b**), Mito-Tracker Deep Red FM (**c**), and Lyso-Tracker Red DND (**d**) in HeLa cells pretreated with TPCI (1 μM) for 24 h. **e-f**, Colocalization of TPCI@Lipo and Golgi-Tracker Red (**e**), ER-Tracker Red (**f**), Mito-Tracker Deep Red FM (**g**), and Lyso-Tracker Red DND (**h**) in HeLa cells pretreated with TPCI@Lipo (TPCI, 1 μM) for 24 h. Scale bar: 15 μm. The fluorescence signals of TPCI were shown in green, and the fluorescence signals of organelle trackers were shown in red. The fluorescence signals of TPCI and organelle trackers along the selected lines were shown on the right (indicated by a yellow line in each image).


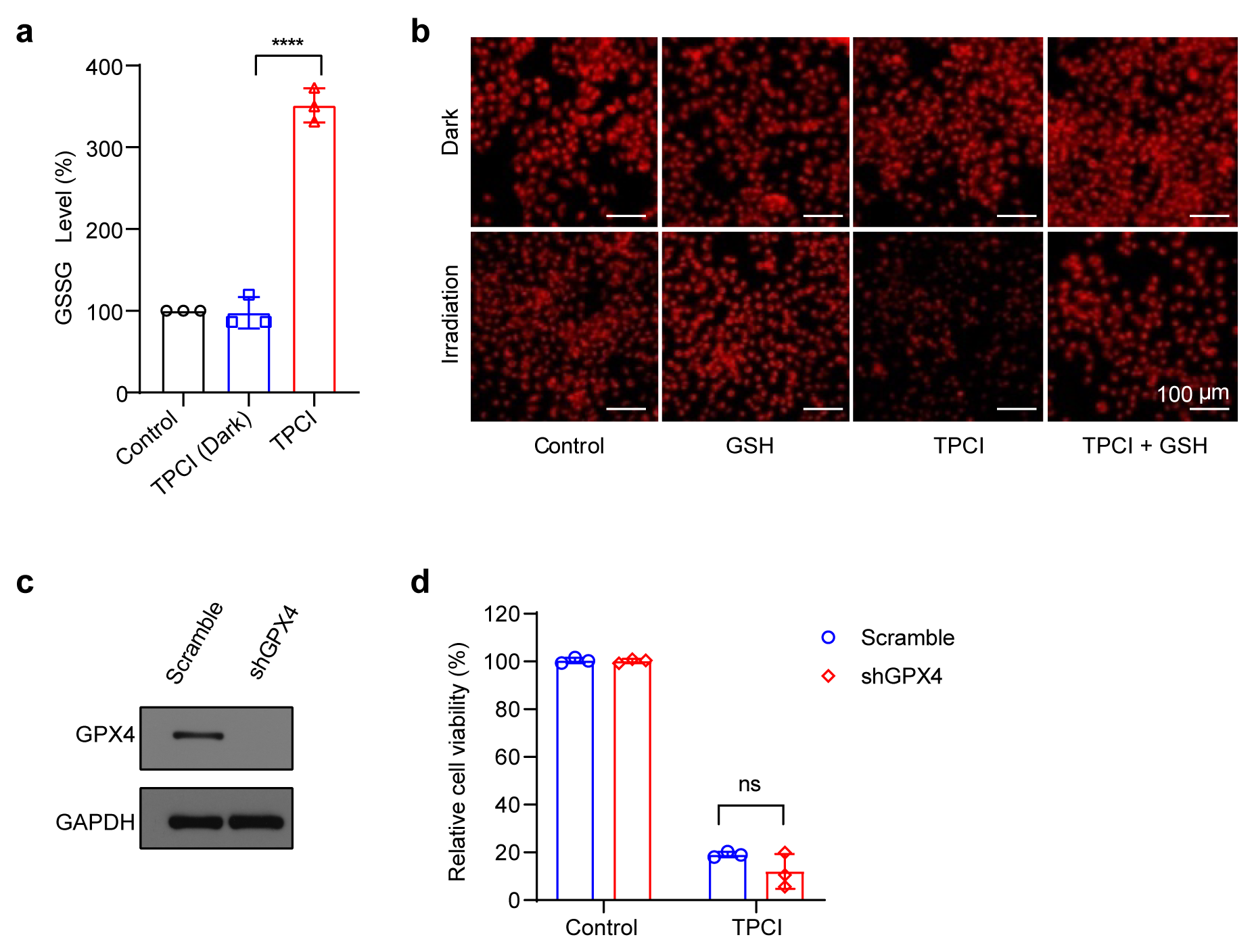


**Supplementary Fig. 6. a**, GSSG levels of HeLa cells receiving TPCI treatment (The data were from 3 independent biological replicates). **b**, Fluorescence images of HeLa cells receiving TPCI treatment. HeLa cells were stained with BODIPY^581/591^ C11 (5 μM), and treated with or without GSH (4 mM). **c**, Knockdown of GPX4 by shRNA (shGPX4) in HeLa cells. **d**, Relative viability of the scramble and GPX4-knockdown HeLa cells treated by TPCI and light irradiation. TPCI concentration was 1 μM; Irradiation conditions: 460 nm, 1 mW cm^-2^, 20 min. The data were shown as mean ± SD from 3 independent biological replicates. Statistical significance was analyzed by two-tailed unpaired Student's t-test (ns: no significance, and *****P* < 0.0001).

# **3. References**
